# A Novel Bifunctional Alkylphenol Anesthetic Allows Characterization of γ-Aminobutyric Acid, Type A (GABA_A_), Receptor Subunit Binding Selectivity in Synaptosomes*[Fn FN2]

**DOI:** 10.1074/jbc.M116.736975

**Published:** 2016-07-26

**Authors:** Kellie A. Woll, Sruthi Murlidaran, Benika J. Pinch, Jérôme Hénin, Xiaoshi Wang, Reza Salari, Manuel Covarrubias, William P. Dailey, Grace Brannigan, Benjamin A. Garcia, Roderic G. Eckenhoff

**Affiliations:** From the Departments of ‡ Anesthesiology and Critical Care and; § Pharmacology and; the ‡‡ Epigenetics Program, Department of Biochemistry and Biophysics, University of Pennsylvania Perelman School of Medicine, Philadelphia, Pennsylvania 19104,; the ¶ Center for Computational and Integrative Biology and; §§ Department of Physics, Rutgers University, Camden, New Jersey 08102,; the ‖ Department of Chemistry, University of Pennsylvania School of Arts and Sciences, Philadelphia, Pennsylvania 19104,; the ** Laboratoire de Biochimie Théorique, Institut de Biologie Physico-Chimique, CNRS UMR 8251 and Université Paris Diderot, 5013 Paris, France, and; the ¶¶ Department of Neuroscience and Farber Institute for Neuroscience, Sidney Kimmel Medical College, Thomas Jefferson University, Philadelphia, Pennsylvania 19107

**Keywords:** anesthesia, anesthetic, click chemistry, GABA receptor, photoaffinity labeling

## Abstract

Propofol, an intravenous anesthetic, is a positive modulator of the GABA_A_ receptor, but the mechanistic details, including the relevant binding sites and alternative targets, remain disputed. Here we undertook an in-depth study of alkylphenol-based anesthetic binding to synaptic membranes. We designed, synthesized, and characterized a chemically active alkylphenol anesthetic (2-((prop-2-yn-1-yloxy)methyl)-5-(3-(trifluoromethyl)-3*H*-diazirin-3-yl)phenol, AziP*m-click* (1)), for affinity-based protein profiling (ABPP) of propofol-binding proteins in their native state within mouse synaptosomes. The ABPP strategy captured ∼4% of the synaptosomal proteome, including the unbiased capture of five α or β GABA_A_ receptor subunits. Lack of γ2 subunit capture was not due to low abundance. Consistent with this, independent molecular dynamics simulations with alchemical free energy perturbation calculations predicted selective propofol binding to interfacial sites, with higher affinities for α/β than γ-containing interfaces. The simulations indicated hydrogen bonding is a key component leading to propofol-selective binding within GABA_A_ receptor subunit interfaces, with stable hydrogen bonds observed between propofol and α/β cavity residues but not γ cavity residues. We confirmed this by introducing a hydrogen bond-null propofol analogue as a protecting ligand for targeted-ABPP and observed a lack of GABA_A_ receptor subunit protection. This investigation demonstrates striking interfacial GABA_A_ receptor subunit selectivity in the native milieu, suggesting that asymmetric occupancy of heteropentameric ion channels by alkylphenol-based anesthetics is sufficient to induce modulation of activity.

## Introduction

γ-Aminobutyric acid (GABA) is well established as the major inhibitory neurotransmitter within the adult mammalian brain. The majority of GABA inhibitory activity is a consequence of binding to a set of pentameric ligand-gated ion channels called the GABA type A (GABA_A_) receptor. GABA_A_ receptors are largely heteromeric protein complexes composed of five different subunits that form a central pore that mediates chloride flux. Including the multiple isoforms, heterogeneity of the receptor is extensive with potentially more than 800 combinations (1). This complexity can be partially simplified by the degrees of selective cellular localization for some subunits and isoforms. Synaptic GABA_A_ receptors contribute considerably to the communication between neurons, including influencing presynaptic neurotransmitter release as well as inducing postsynaptic hyperpolarization (1–4). Numerous studies have indicated that synaptic GABA_A_ receptors are predominantly of a 2α:2β:1γ stoichiometry (5, 6) that organizes in an alternating order (*e.g.* γαβαβ anti-clockwise as seen from synaptic cleft) (5–7). The resulting complex yields an abundance of potential ligand interaction surfaces within one heteropentamer, including at least four unique subunit interfaces. As such, it is justified that the composition and orientation of subunits are functionally significant, with different pharmacological properties pertaining to different GABA_A_ receptor complexes (1, 8).

**FIGURE 1.**
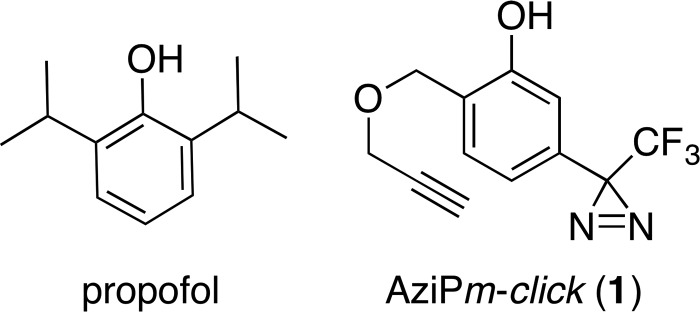
**Clickable photoactive propofol analogue.** Chemical structures of propofol and AziP*m-click* (**1**).

Numerous drugs influence GABA_A_ receptor activity, including general anesthetics that are used extensively in modern medicine and in scientific research (9). For example, 2,6-diisopropylphenol (propofol^2^ ( (Fig. 1) has been strongly implicated as a modulator of the GABA_A_ receptor. Relatively low concentrations of this alkylphenol significantly potentiate GABA-induced current, an action that hyperpolarizes the post-synaptic membrane and thereby likely contributes to hypnosis and possibly other anesthesia phenotypes (10, 11). Furthermore, multiple reports indicate that phasic inhibition is particularly sensitive to low concentrations of propofol, suggesting that synaptic GABAergic signaling is a critical pathway for the anesthetic's pharmacological effects (12–14).

Investigations have focused on the potential binding sites within heterologously expressed αβγ GABA_A_ receptors. A wide range of mutagenesis studies have probed ligand-gated ion channel electrophysiology and have shown that mutation of various residues predicted to reside within subunit interfacial regions alters propofol modulation (9, 15–17). Particular point mutations within β subunits, such as Asn-265, greatly decreased propofol-positive modulation (11, 18). Our previous work using the tritiated photoaffinity ligand (PAL) *meta*-azipropofol demonstrated frequent labeling of interfacial residues within the heterologously expressed Cys loop superfamily of receptors, including α_1_β_3_γ_2_ GABA_A_ receptors (19). These findings further suggest that subunit interfaces are potentially involved in propofol modulation. Structure-activity relationships applying alkylphenol analogues and/or other chemical derivatives (20, 21), molecular dynamic (MD) simulations (22, 23), as well as other investigations have suggested complex physicochemical interactions between propofol and GABA_A_ receptors (24). Together, these studies have provided insight regarding the potential mechanism by which propofol perturbs GABA_A_ receptor protein dynamics. However, in addition to the biased nature of using heterologously expressed receptors, it is recognized that each method has experimental limitations that result in the current uncertainty regarding alkylphenol interactions within the receptor.

Our objective was to advance the current understanding of anesthetic interactions with heteromeric receptors by addressing the interaction(s) of alkylphenols with GABA_A_ receptors within their native synaptic milieu. Our approach applied a novel chemically active alkylphenol anesthetic for quantitative affinity-based protein profiling (ABPP) of propofol within synaptosomes. By utilizing a native tissue-derived system, the relative GABA_A_ receptor subunit expression, pentameric composition, protein-protein interactions, and lipid milieu are maintained. We assessed these binding results with independent MD simulations using the alchemical free energy perturbation (AFEP) algorithm (25) to predict potential molecular recognition elements within α_1_β_3_γ_2_ GABA_A_ receptor-binding sites. Finally, we examined the impact of these molecular recognition elements within the synaptic GABA_A_ receptors with photoaffinity protection experiments. Our studies led to the unbiased identification of GABA_A_ receptor subunits in native synaptic membranes as alkylphenol-binding proteins. Our investigation suggested higher affinity interactions for β+/α− and α+/β− interfacial sites relative to γ-containing subunit interfaces and hydrogen bonding as the major recognition element for the alkylphenol-GABA_A_ receptor complex.

## Results

### 

#### 

##### Synthesis of AziPm-click (**1**)

To identify the alkylphenol-binding proteins within the synaptic proteome, we developed 2-((prop-2-yn-1-yloxy)methyl)-5-(3-(trifluoromethyl)-3*H*-diazirin-3-yl)phenol, or AziP*m-click* (**1**), a photoaffinity tandem bioorthogonal alkylphenol anesthetic ligand (Fig. 1). AziP*m-click* (**1**) was designed to integrate two chemically active groups that allow for ABPP as follows: 1) a diazirine photoreactive group to covalently label protein interaction sites, and 2) an alkynyl group for covalent attachment of a reporter tag by 1,3-dipolarcycloaddition reaction (*e.g.* “Click Chemistry”) to capture and identify photoaffinity labeled proteins within the synaptic proteome.

**SCHEME 1:**
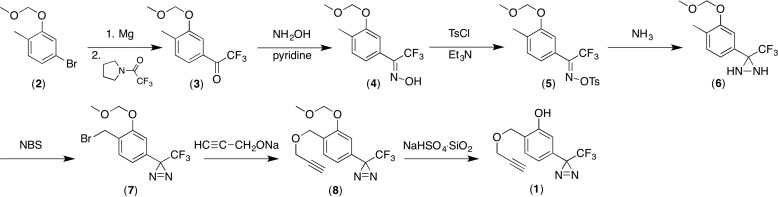


Synthesis of AziP*m-click* (**1**), shown in Scheme 1 (described in supplemental S2–S7), starts with the previously reported 4-bromo-2-(methoxymethoxy)-1-methylbenzene (**2**) (26). Conversion of **2** to the Grignard reagent using magnesium in THF followed by treatment with pyrrolidine trifluoroacetamide produced trifluoromethyl ketone **3**. Conversion of **3** to the oxime **4** and oxime tosylate **5** followed standard procedures. Treatment of **5** with excess liquid ammonia produced diaziridine **6** that was oxidized to the diazirine **7** using pyridinium dichromate. Benzylic bromination using *N*-bromosuccinimide produced **8,** which was treated with the sodium salt of propargylic alcohol in tetrahydrofuran to provide **9**. Removal of the methoxymethyl protecting group in the presence of the propargylic ether required carefully controlled conditions and was finally accomplished using sodium hydrogen sulfate-impregnated silica gel in methylene chloride (27).

##### Physicochemical and Protein-binding Properties

**FIGURE 2.**
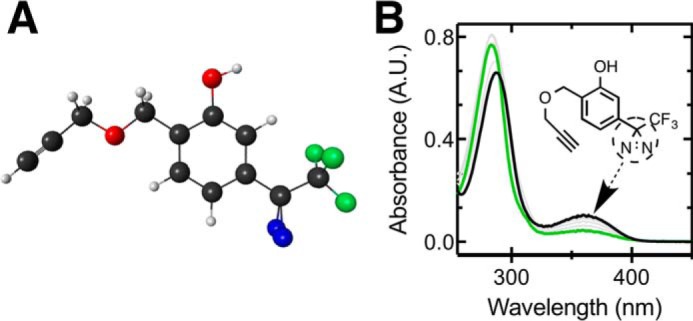
**AziPm-*click* (1) geometry and photoreactivity.** *A, ball and stick* structure of AziPm-*click* (**1**) in predicted lowest energy conformation (*gray,* carbon; *red,* oxygen; *blue,* nitrogen, *green,* fluorine). *B,* UV absorption spectra of AziP*m-click* (**1**) (175 μm) in double distilled water (*black line*) over the course of UV irradiation time points (*gray* and *green lines*).

The physicochemical characteristics of propofol and AziP*m-click* (**1**) are summarized in Table 1, and the geometry-optimized structure is shown in Fig. 2*A*. The UV absorption spectrum of AziP*m-click* (**1**) shows a well defined peak between 330 and 400 nm due to the diazirine group (methanol extinction coefficient (Σ_365 nm_) of 580 m^−1^ cm^−1^). Over the course of UV irradiation using a Rayonet RPR-3500 lamp within the aqueous solution, the AziP*m-click* (**1**) diazirine absorbance band decreased intensity indicating photoactivation (Fig. 2*B*). The time-dependent photoreactivity of AziP*m-click* (**1**) in aqueous solution was a single exponential decay with a half-life (*t*_½_) of 25 min (95% CI; 20–33) within a 1-cm path length cuvette and 6 cm from the lamp. To confirm retention of other major molecular recognition features, we compared equilibrium binding affinities of applied alkylphenol general anesthetics with the model protein apoferritin by isothermal calorimetry and 1-aminoanthracene (1-AMA) competition (28, 29). The affinities of alkylphenols for apoferritin have shown to be well correlated with GABA_A_ receptor potentiation (30–32); results are summarized in Table 2.

**TABLE 1:** **Physicochemical properties**

	Mass	Density	clogP
	*Da*	*g/ml*	
AziP*m-click* (**1**)	270	1.19	3.55
propofol	178	0.96	3.79

**TABLE 2:** **Equilibrium binding parameters** ITC is isothermal calorimetry.

	Propofol		AziP*m-click* (1)	
	ITC	1-AMA displacement*^a^*	ITC	1-AMA displacement*^a^*
*K_D_* (95% CI; μ*m*)	9 (7.1–11)	2.4 (1.3–4.4)	22 (20- 24)	4.0 (1.8–8.7)
Hill slope (mean ± S.E.)	1*^b^*	−1.1 ± 0.33	1*^b^*	−0.97 ± 0.39

*^a^ K_D_* indicates fluorescence data derived from the Cheng-Prusoff equation.

*^b^* Stoichiometry of HS*a*F sites was modeled for one site; therefore, the Hill slope is fixed at 1.

##### Fluorescent Profiling of Alkylphenol-binding Proteins

**FIGURE 3.**
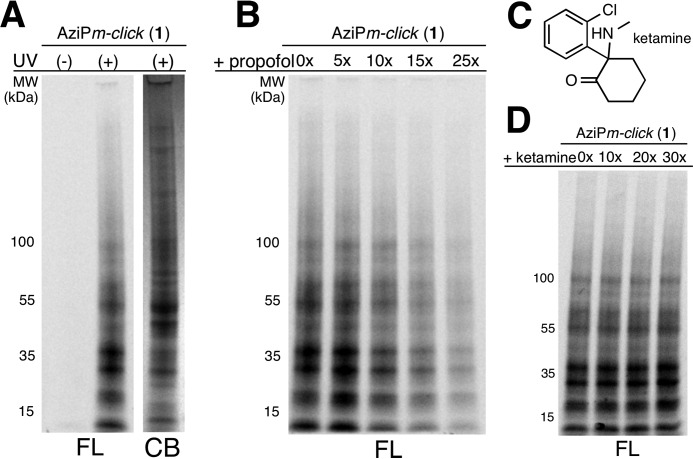
**Fluorescent profiling of propofol proteome.** *A,* fluorescent image (*FL*) of SDS-polyacrylamide gel of synaptosomes exposed to AziPm-*click* (**1**) with or without UV irradiation and corresponding Coomassie Blue (*CB*) stain of UV-irradiated synaptosomes. *B,* protection from AziPm*-click* (**1**) labeling of synaptosomes by propofol at 5× (75 μm), 10× (150 μm), 15× (225 μm), and 25× (375 μm). *C,* chemical structure of ketamine. *D,* protection from AziPm-*click* (**1**) labeling of synaptosomes by ketamine at 10× (150 μm), 20× (300 μm), and 30× (450 μm). All experiments were conducted in triplicate.

To confirm the functionality of both the chemically active groups for downstream ABPP, we employed AziP*m-click* (**1**) within mouse synaptosomes using an azide-PEG3-Alexa 488 fluorophore as a reporter tag. The fluorescent labeling of proteins was reliant on UV exposure (Fig. 3*A*). Fluorescent labeling was decreased with increased concentrations of propofol indicating protection of alkylphenol-binding proteins within synaptosomes (Fig. 3*B*). To control for potential “inner filter” of UV light, ketamine was employed as a protecting ligand (Fig. 3*C*), which conferred no changes in fluorescence intensity seen in Fig. 3*D*.

##### α_1_β_2_γ_2L_ GABA_A_ Receptor Electrophysiology with AziPm-click (**1**)

**FIGURE 4.**
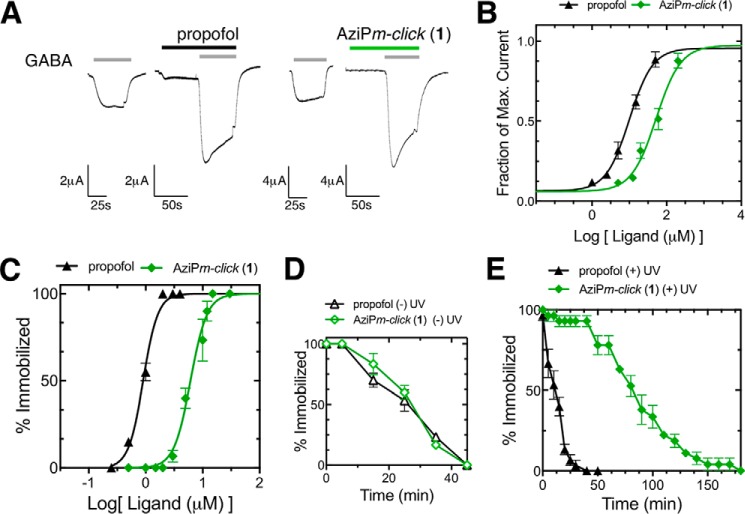
**α_1_β_2_γ_2L_ GABA_A_ receptor and anesthetic activity of AziP*m-click* (1).** *A,* representative traces of ligand activity on heterologously expressed α_1_β_2_γ_2L_ GABA_A_ receptors in *X. laevis* oocytes. *Traces* are shown with the oocyte responses to GABA EC_10_ value and corresponding modulation propofol (3 μm) or AziPm-*click* (**1**) (20 μm). *B,* concentration-response curves for propofol (*black circle*) and AziP*m-click* (**1**) (*green diamond*) for the positive modulation of heterologously expressed GABA_A_ receptor α_1_β_2_γ_2L_ in *X. laevis* oocytes. Each point represents the mean of four oocytes (*n* = 4) ± S.E., and data were fitted to a sigmoidal dose-response curve with variable Hill slope. *C,* dose-response curves for propofol (*n* = 210; *black circle*) and AziP*m-click* (**1**) (*n* = 300; *green diamond*) for loss of spontaneous movement in tadpoles. Data were fitted to a sigmoidal dose-response curve with variable Hill slope, and the EC_50_ and Hill slope values are represented in Table 3. *D,* time course of recovery control for *X. laevis* tadpoles following propofol (*n* = 30; *black open circle*) or AziP*m-click* (**1**) (*n* = 30; *green open diamond*) equilibration and 10 min no UV treatment. *E,* time course of recovery for tadpoles following propofol (*n* = 30; *black filled circle*) or AziP*m-click* (**1**) (*n* = 30; *green filled diamond*) equilibration and 10 min of low intensity UV irradiation.

AziP*m-click* (**1**) was functionally active on α_1_β_2_γ_2L_ GABA_A_ receptors heterologously expressed in *Xenopus* oocytes. AziP*m-click* (**1**) demonstrated similar positive modulation activity as propofol (Fig. 4, *A* and *B*). The EC_50_ value for propofol-positive modulation (at a GABA EC_10_) in our system was 10 μm (95% CI; 3.3–17). AziP*m-click* (**1**) required a higher concentration for a similar response with an EC_50_ of 49 μm (95% CI; 38–61).

##### In Vivo Anesthetic Activity and Photoaffinity Labeling

Propofol and AziP*m-click* (**1**) demonstrated similar pharmacological end points within *Xenopus laevis* tadpoles, inducing reversible hypnosis with no observable toxicity summarized in Table 3 and shown in Fig. 4*C*. To indicate photoaffinity labeling of pharmacologically relevant targets, we demonstrated that AziP*m-click* (**1**) produced sustained anesthetic end points (immobility) *in vivo* after UV irradiation (33). *X. laevis* tadpoles were exposed to 12 μm AziP*m-click* (**1**) or 3 μm propofol for 30 min. Tadpoles were then exposed to 10 min of low intensity UV irradiation or were maintained as a 10-min non-UV irradiation control. Similar to our previous reports for *meta*-azipropofol (33), only tadpoles exposed to AziP*m-click* (**1**) and 10-min UV irradiation displayed prolonged immobility after drug washout (Fig. 4, *D* and *E*).

**TABLE 3:** **Tadpole studies**

	EC_50_ (95% CI; μ*m*)	Hill slope (mean ± S.E.)
AziP*m-click* (**1**)	6.1 (5.1–7.4)	3.0 ± 0.54
Propofol	0.90 (0.84–0.97)	3.4 ± 0.31

##### ABPP for Alkylphenol Anesthetics

**FIGURE 5.**
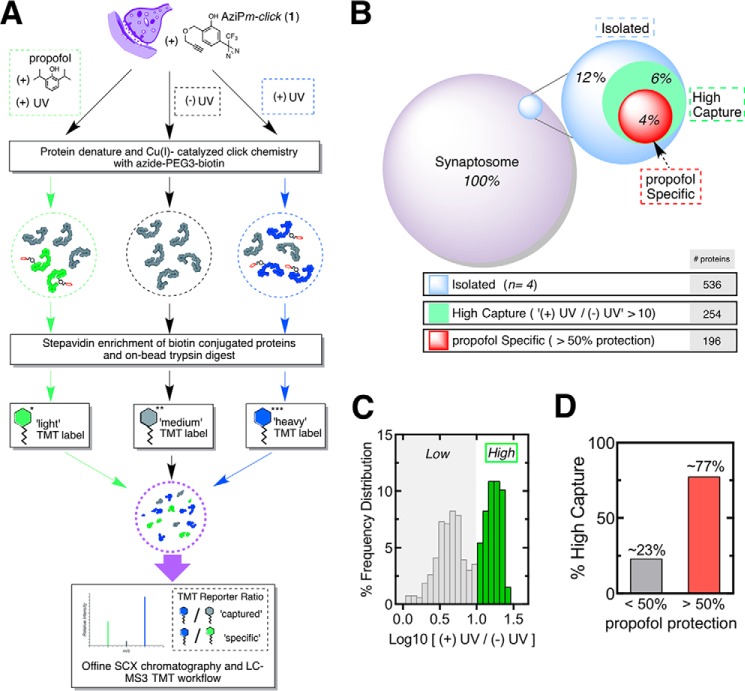
**Affinity-based propofol profiling of alkylphenol-binding proteins in native synaptosomes.** *A,* scheme for capture and analysis of AziP*m-click* (**1**) labeling profiles in synaptosomes by biotin-streptavidin methods, TMT, labeling for relative quantification, strong cation exchange chromatography (*SCX*), and Nanoliquid chromatography-three-stage mass spectrometry (NanoLC-MS3) analysis. *B,* distribution of protein groups for the AziP*m-click* (**1**) capture and approximate percentage of full synaptosomal proteome, with a summary of the group's threshold requirements. Proteomic experiments were conducted in quadruplicate; the log2 standard deviation between datasets was calculated as 0.28 for heavy over intermediate TMT-labeled samples and 0.17 for heavy over light TMT-labeled samples. *C,* TMT ratio frequency distribution (log10 scale) of UV *versus* no UV irradiation with high capture efficiency threshold. *D,* percent of high capture group proteins that demonstrated less than or greater than 50% protection by propofol.

The ABPP workflow using AziP*m-click* (**1**) with relative quantification is summarized in Fig. 5*A*. Azide-PEG3-biotin was employed as the reporter tag for streptavidin-affinity isolation of photoaffinity labeled protein targets. Tandem mass tag (TMT) isotopic labeling and three-stage mass spectrometry (MS3) (34) were coupled to ABPP for quantitative assessment of capture and propofol protection. The totals for identified proteins are summarized in Fig. 5*B*. The AziP*m-click* (**1**) proteome contained a discernible group of proteins that demonstrated a high degree of capture efficiency with a greater than 10 enrichment factor (Fig. 5*C*). Of the higher capture group, the majority of proteins displayed propofol specificity with a greater than 50% protection and a decrease of at least 5 in enrichment factor (Fig. 5*D* and supplemental Table 1). Five GABA_A_ receptor subunits (α_1,3_ and β_1–3_) were identified as propofol-specific proteins. All subunits showed a decrease of at least 10 in enrichment factor with propofol protection. Based on experimental studies in heterologous expression systems, the GABA_A_ receptor is considered to be an important alkylphenol target. This unbiased ABPP capture of the receptor from a complex biological milieu, derived from native tissue, is to our knowledge the first such demonstration, and it further validates the receptor as a pharmacologically relevant target. To further corroborate the ABPP results of our approach, we examined the apparent subunit-level selectivity binding to this single target with other approaches.

##### MD Simulations of Dynamic Propofol Interactions with α_1_β_3_γ_2_ GABA_A_ Receptor

**FIGURE 6.**
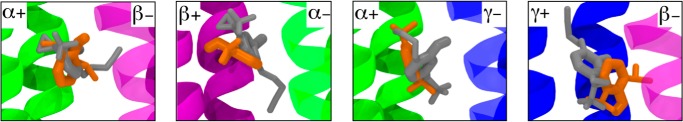
**Intersubunit propofol and AziP*m-click* (1) occupancy in an α_1_β_3_γ_2_ GABA_A_ receptor as predicted by AutoDock Vina simulations.** Helices of the four distinct subunit interface pairs (α_1_, *green*; β_3_, *magenta*; γ_2_, *blue*) with the highest scored docking poses for propofol (*orange*) and AziP*m-click* (**1**) (*gray*).

To understand the apparent subunit specificity noted in the above experiments, MD simulations for alkylphenol anesthetic binding were generated with an α1β3γ2 GABA_A_ model derived from an α1β1γ2 GABA_A_ model used previously (35). Docking calculations to the entire pentamer identified at least one propofol pose in each subunit interface, β+/α− (two sites), α +/β−, α+/γ−, and γ+/β−, as shown in Fig. 6*A*. Other than the channel lumen, no alternate sites were consistently detected over multiple docking runs. Docking of AziP*m-click* (**1**) to the same model yielded overlapping sites, demonstrating that AziP*m-click* (**1**) is not sterically hindered from binding to the intersubunit sites, despite the larger molecular size. Furthermore, as shown in Fig. 6, AziP*m-click* docking simulations yield similar orientations to propofol suggesting common favorable interactions.

**FIGURE 7.**
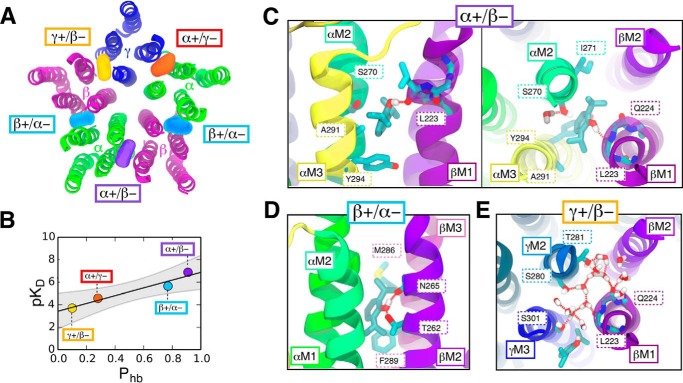
**Selectivity of intersubunit propofol binding in an α_1_β_3_γ_2_ GABA_A_ receptor as predicted by molecular dynamics simulations using the AFEP algorithm.** *A,* five propofol molecules (colored surfaces) docked in the GABA_A_ receptor subunit interfaces (β+/α− (−2 sites)) are as follows: *cyan,* α+/β−; *violet,* α+/γ−; *red,* γ+/β−, *orange*. The transmembrane domain is viewed from the extracellular side along the pore axis and colored by subunit type; α_1_, *green*; β_3_, *magenta*; and γ_2_, *blue. B,* computational results for propofol p*K_D_* and its likelihood of hydrogen bonding to protein cavity residues (*P*_hb_) can be well fit by the line p*K_D_* = *a* (*P*_hb_) + *b*, where *a* = 3.4 ± 0.8 and *b* = 3.4 ± 0.1, and the 95% confidence band is shown in *gray. C–E,* interactions of propofol and water in the high affinity and low affinity interfacial sites. Hydrogen bonds, *red dashed lines. C,* propofol binding in α+/β− interface that contained seven polar residue side chains (*left,* side view; *right, top view*) forms a persistent hydrogen bonding with a backbone carbonyl group exposed by the M1 helical bulge (βLeu-223). *D,* bound propofol at the β+/α− interfacial site, which contained seven polar residue side chains, (side view) alternates between hydrogen bonds to β+M2:Thr-262 and β+M2:Asn-265. For compactness, the image shows a rare frame in which both hydrogen bonds coexist. *E,* in the γ+/β− interface eight polar residue side chains were present (*top view*); these residues favor hydrogen bonding with a water cluster stabilized by polar residues γ+Thr-281 and γ+Ser-301, which are homologous to hydrophobic residues in α and β subunits (see Fig. 7).

A receptor-propofol complex was constructed with one propofol molecule in the highest scoring pose for each subunit interface (Fig. 7*A*). The complex was embedded in a fully hydrated phosphatidylcholine membrane and simulated for 270 ns using traditional equilibrium MD with atomic resolution. In addition to allowing the propofol in the intersubunit space to equilibrate before the affinity calculations, we used this simulation to characterize and compare the microscopic interactions between propofol and the binding pocket across subunits.

At the conclusion of the traditional MD simulation, standard binding affinities for propofol in each of the four distinct sites were calculated using separate 24-ns AFEP simulations. The AFEP method also involves running MD simulations but is designed to facilitate simultaneous calculation of average quantities appearing in the Zwanzig equation (36), an exact expression for the free energy difference between two states (*e.g.* bound and unbound) that inherently accounts for all entropic and enthalpic contributions. The results from the AFEP simulations indicate three higher affinity sites at the α+/β− and two β+/α− interfaces, with *K_D_* values similar to propofol EC_50._ *K_D_* values for the α+/γ− and +γ/β− interfaces, however, suggest markedly weaker propofol binding to those sites (Table 4).

**TABLE 4:** **Binding affinities of propofol bound to one of four GABA_A_ receptor interfacial sites (shown in Fig. 5, interfaces notated counter-clockwise), calculated using AFEP**

Interface	*K_D_*	*K_D_ e*^−δ/*RT*^ − *K_D_ e*^δ/*RT*^ *^a^*
	μ*m*	μ*m*
α+/β−	0.1	0.02–0.7
β+/α−	2.0	0.4–10
α+/γ−	30	5–200
γ+/β−	200	40–1000

*^a^ K_D_* range corresponds to an uncertainty in Δ*G* of δ = 1 kcal/mol. Challenges inherent in determining constants required for correction to laboratory conditions contribute significantly to δ; errors in relative values of *K_D_* are substantially reduced compared with those for absolute *K_D_*.

**FIGURE 8.**
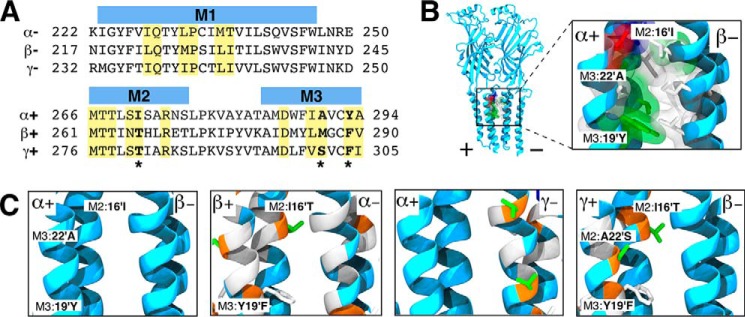
**Sequence variation in interfacial binding sites of an α_1_β_3_γ_2_ GABA_A_ receptor heteropentamer.** *A,* sequence alignment of + and − subunit interfaces that contribute to the formation of interfacial binding sites. Highlighted residues represent residue side chains that directly contribute to the formation of the binding cavity. *Bold* and * residues denote key sequence variations in the interfacial binding sites. *B* and *C,* helices of the four distinct subunit interface pairs with α+/β− interface as the reference pair. In all panels, side chains are colored by residue type as follows: polar (*green*), hydrophobic (*white*), acidic (*red*), and basic (*blue*). *B,* extended view and binding site cavity view of the α+/β− interface reference pair with all cavity contributing side chain residues represented. *C,* helices of the four distinct subunit interface pairs are colored according to sequence differences with the α+/β− interface as the reference subunit pair displaying identical (*light blue*), similar (*white*), and change in residue type (*orange*). Note that for a given interface, coloring of the + and − subunit backbone reflects sequence differences from α_1_ and β_3_, respectively. Cavity residues are labeled according to a prime-numbering system in which M2:16′ is equivalent to Ile-271, Thr-266, and Thr-281 for α1, β_3_, and γ_2_ subunits, respectively; M3:19′ is Tyr-294, Phe-289, and Phe-304 and M3:22′ is Ala-291, Met-286, and Ser-301 with the same ordering.

The particularly low affinity of propofol for the +γ/β− interfacial cavity, which has one more polar residue than the other interfacial cavities (Fig. 8*A*), seemed potentially contradictory to an essential role for hydrogen bonding. As shown in Fig. 7*B*, however, the p*K_D_* values for different subunit interfaces were found to be strongly correlated (*r*^2^ = 0.94) with the probability (*P*_hb_) that the propofol hydroxyl would form at least one hydrogen bond with one of the cavity-lining residues. Propofol in either of the two sites with low *K_D_* values (α+/β− and β+/α−) had at least *P*_hb_ >0.8; for the two low affinity sites, this probability was significantly reduced (*P*_hb_ <0.3). Thus, although propofol affinity is correlated with propofol hydrogen bonding, propofol is less likely to form hydrogen bonds with the more hydrophilic +γ/β− interfacial cavity. This result was due to stable hydration of the +γ/β− cavity, due to interactions of water molecules with γSer-301 and γThr-281 (Fig. 7*E*). The water molecules compete for hydrogen bonding partners and interact unfavorably with the propofol isopropyl groups.

Within the highest affinity site at the α+/β− interface, propofol orients as a hydrogen donor to the carbonyl backbone of Leu-223 within the βM1 transmembrane helix (Fig. 7*C*) where a bulge in backbone hydrogen bonding is observed in crystal structures for both GluCl (37) and the GABA_A_ receptor β_3_ homopentamer (38). Similar behavior was observed in simulations of triiodothyronine bound to interfacial sites (39). In the β+/α− interface, propofol alternates rapidly between serving as a hydrogen acceptor for βM2:Thr-262 and donor for βM2:Asn-265 (Fig. 7*D*). The associated slight reduction in p*K_D_* is consistent with the slight reduction in *P*_hb_ and the line of best fit.

The AFEP calculations yield an intermediate affinity of propofol for the γ−/α+ interfacial site. Residues of the γ-face, however, are nearly identical to those of the β-face, as shown in Fig. 8, *B* and *C*, and sequence differences among site residues are unable to account for the moderate differences in hydrogen bonding and affinity between γ−/α+ and the higher affinity β−/α+ site. Because hydrogen bonding of propofol to the M1 backbone is frequently observed for β− but not γ−, it is possible that sensitivity of fluctuations in M1 secondary structure to non-cavity residues causes the observed weak sequence dependence. If so, the result suggests a further uncertainty in interpretations of mutagenesis experiments and the underlying assumption that identified residues are contact residues.

##### Propofol and 2-Fluoro-1,3-diisopropylbenzene (Fropofol) Protection of GABA_A_ Receptor Subunits

**FIGURE 9.**
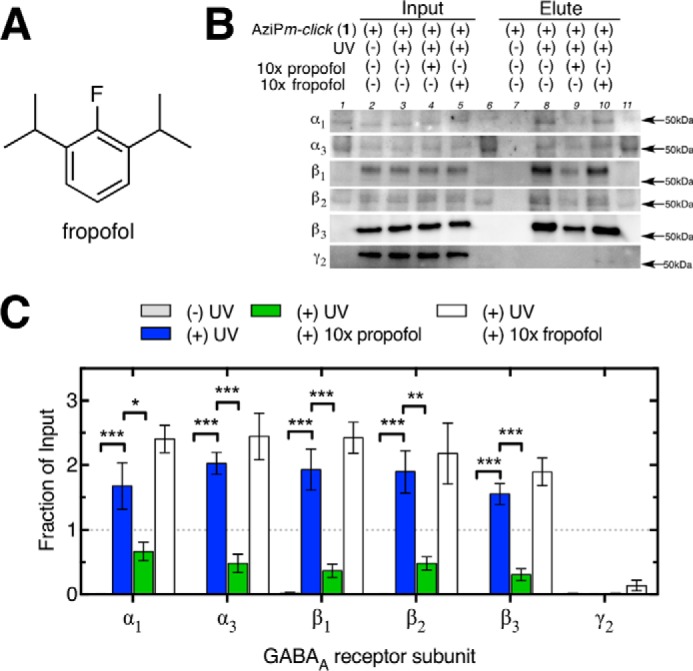
**Ligand protection of synaptic GABA_A_ receptor capture.** *A,* chemical structure of fropofol. *B,* representative Western blots for GABA_A_ receptor subunits of input (*lanes 2–5*) and the corresponding elution (*lanes 7–10*) for synaptosomal samples exposed to AziP*m-click* (**1**) (10 μm) with or without UV irradiation and with or without co-exposure with propofol (100 μm) or fropofol (100 μm). *Lanes 1, 6,* and *11* contain protein ladders. *B,* comparison of non-UV and UV capture with or without propofol or fropofol protection for each GABA_A_ receptor subunit; values are represented as the mean of four experiments ± S.E. of the fraction of the corresponding input sample. Data were analyzed by two-way analysis of variance with Tukey's multiple comparison test comparing fraction captured between protection conditions for each subunit. Significant differences compared with UV-irradiated eluate preparation without protection ligand are shown (***, *p* < 0.001; **, *p* < 0.01; *, *p* < 0.05).

To experimentally evaluate the role of the alkylphenol hydroxyl in selective binding to sites within synaptic GABA_A_ receptor subunits, we applied the fluorine-substituted analogue fropofol (Fig. 9*A*) within protection experiments (40). Previously, fropofol did not modulate or disrupt propofol potentiation of the GABA_A_ receptor, and it did not cause immobilization at even 100-fold higher concentrations than propofol. In contrast, fropofol did display similar binding as propofol to protein sites that were not dependent on hydrogen bond interactions (40). Azide-PEG3-biotin was employed as the reporter tag for streptavidin-affinity isolation of protein targets photoaffinity labeled by AziP*m-click* (**1**) with or without protection ligands propofol or fropofol. Protein levels of GABA_A_ receptor subunits were determined by Western blotting before (or “input”) and after (or “elute”) streptavidin capture of biotinylated proteins. All GABA_A_ receptor subunits were detected within synaptosomes prior to capture (Fig. 9*A*), consistent with synaptic localization of these subunits. After capture, only α and β subunits were detected. All GABA_A_ receptor α and β subunits showed significant decreases in capture efficiency when propofol was present during UV irradiation. Unlike propofol, fropofol was unable to protect GABA_A_ receptor α and β subunits from capture (Fig. 9, *A* and *B*).

## Discussion

In this study we investigated the molecular mechanisms and placement of alkylphenol anesthetic binding to synaptic GABA_A_ receptors. This multifaceted study integrated an assessment of total synaptic GABA_A_ receptor binding relative to the native proteome with a targeted investigation of key molecular recognition elements that contribute to binding affinity. Our approach deployed a novel anesthetic PAL containing a click chemistry moiety for downstream quantitative ABPP, as well as AFEP MD simulations of receptor binding and photoaffinity protection experiments. Together, these studies add to the understanding of propofol pharmacology and the dynamic behaviors of heteropentameric receptors.

Previous work has shown that the ligand-gated receptors, GABA_A_ receptors in particular, are modulated by propofol. Site-directed mutagenesis within heterologous expression systems and animal models provides the advantage of demonstrating drug activity and pharmacological changes as well as candidate regions of drug binding. The recognized pitfalls of mutagenesis, like the inherent ambiguity of defined interaction regions, perturbation of native protein dynamics and/or genetic compensation, mandates that additional strategies be deployed to complement these investigations. Photoaffinity labeling has been one method used to further the understanding of anesthetic-binding sites (41). Multiple PALs for propofol have been reported (42, 43), including one from our own laboratory (29), and have furthered an understanding of propofol-protein interactions. Previous photoaffinity labeling studies, however, have had their own recognized limitations, particularly reliance on heterologous overexpression and reconstituted systems.

### 

#### 

##### GABA_A_ Receptor Binding of Alkylphenol-based Anesthetics within the Synaptic Proteome

To respond to these issues, we developed a photoaffinity tandem bioorthogonal ligand, AziP*m-click* (**1**) to evaluate the alkylphenol-based anesthetic-binding proteins within the native synaptosomal proteome using ABPP. Subunits of the GABA_A_ receptor were identified as AziP*m-click* (**1**)-binding proteins that were shared with propofol. To our knowledge the ABPP strategy has provided the first evidence of direct interaction of alkylphenol anesthetics with native tissue-derived synaptic GABA_A_ receptors.

We observed that only selected subunits (α and β) within the heteropentameric receptor were identified as AziP*m-click* (**1**)-binding proteins. No γ subunits were captured in the ABPP experiment despite the fact that the γ_2_ subunit is estimated to incorporate within 75–80% of all receptor complexes (1), act as an important subunit for synaptic GABA_A_ receptor eventual translocation to the plasma membrane (8, 45–47), and was easily identified within our input synaptic milieu. Because AziP*m-click* (**1**) demonstrated hypnotic activity and the ability to act as a positive modulator of the GABA_A_ receptor, the evidence indicates that the orientation(s) of AziP*m-click* (**1**) within the active binding site(s) has the diazirine photoreactive group in close proximity *only* to α and β subunits. The γ_2_ subunit apparently does not contribute surface area to an alkylphenol-binding site at the concentrations used here. When combined with previous work implicating interfacial regions within Cys-loop receptors (22, 48, 49) as propofol-binding sites, we more specifically conclude that alkylphenol occupancy of α and β subunit interfacial sites (*e.g.* β+/α− and α+/β−) is *sufficient* to result in positive modulation.

##### Asymmetrical Propofol Binding to the GABA_A_ Receptor

To evaluate this hypothesis and determine potential elements that mediate propofol affinity, we employed independent MD simulations using rigorous AFEP calculations that focused on propofol interactions within the α_1_β_3_γ_2_ GABA_A_ receptor. Beginning with high scoring poses in the five potential transmembrane sites consistently identified by docking to the full pentamer (all of which are at the subunit interfaces), AFEP calculations yielded *K_D_* values at β+/α− and α+/β− interfaces that were similar to experimental propofol EC_50_ values. In contrast, the *K_D_* values for α+/γ− and γ+/β− interfaces were 1–2 orders of magnitude greater, reaching concentrations of propofol known to be lethal and shown to inhibit the induced positive modulation in electrophysiology studies (50, 51). MD simulations have demonstrated that partial (or asymmetrical) occupancy of the interfacial regions, even in a homomeric pentameric ligand-gated ion channel, has greater effects on pore radius than total (or symmetrical) occupancy (52). Recently, a similar hypothesis was suggested for etomidate and propofol using site-directed mutagenesis of α_1_β_2_γ_2_ GABA_A_ receptor (48). It is also likely that at propofol concentrations where the lowest affinity interfacial sites are occupied, other sites that cause loss of function (*e.g.* pore blocking site(s)) may be occupied as well.

##### Hydrogen Bonding Mediates Alkylphenol-based Anesthetic Binding to the GABA_A_ Receptor

Numerous interactions can contribute to propofol affinity within a protein cavity. These include multiple weak interactions, such as the hydrophobic effect, as well as the potential for stronger interactions, such as hydrogen bonding with the alkylphenol hydroxyl. From the α_1_β_3_γ_2_ GABA_A_ receptor MD simulations, the higher relative affinities for the β+/α− and α+/β− interfaces corresponded with increased probability for hydrogen bond interaction(s) for β+M2 Thr-262 and Asn-265 or β-M1 Leu-223 at a conserved bulge in the M1 backbone. In contrast, the lowest affinity (γ+/β−) interface contains a larger number of potential hydrogen bonding side chains; however, this increases cavity hydration and causes displacement of propofol. Propofol bound to the α+/γ− sites yielded an intermediate affinity and likelihood of hydrogen bonding. Although the α+/γ− side chains are equivalent to those of the higher affinity α+/β− site, we observed that the backbone carbonyl of β− M1 Leu-223 was significantly more likely to serve as a hydrogen bond acceptor than in the homologous residue, γ− M1 Ile-238. The origin of this difference is likely subtle and dependent on any residues that affect M1 secondary structure, not just cavity residues.

To confirm the contribution of the hydroxyl to the alkylphenol-based anesthetic-binding site within captured synaptic GABA_A_ receptor subunits and the functional relevance of these sites, we introduced the fluorine-substituted and therefore hydrogen bond-null ligand fropofol into our investigation. With the fluorine replacement of the alkylphenol hydroxyl, fropofol displayed no activity on heterologously expressed α_1_β_2_γ_2_ GABA_A_ receptors (40). Fropofol does, however, bind to propofol-binding sites that are characterized primarily by the hydrophobic effect (40). We observed that fropofol, unlike propofol, did not protect the α or β subunits from AziP*m-click* (**1**) capture. These results confirm MD simulations that identified hydrogen bonding as a key element contributing to alkylphenol-based anesthetic binding to synaptic GABA_A_ receptors and that such interfacial sites are likely to contribute to the altered functional activity.

##### Study Limitations

Although the goal of this investigation was to reconcile propofol pharmacology with a complex binding proteome, some limitations are implicit in our studies, including points for future improvement. First, our analogue AziP*m-click* (**1**) showed a reduced potency for modulating α_1_β_2_γ_2_ GABA_A_ receptors and a parallel decrease in anesthetic potency as compared with the parent propofol. However, this is well within what has been reported for the alkylphenol chemotype (53), and it is probably a result of the additional bulk and electrostatically active diazirine and ether/alkyne groups, which reduce hydrophobicity. Furthermore, associated docking simulations demonstrated overlapping poses for propofol and AziP*m-click* (**1**). Finally, MD simulations using the AFEP algorithm use the parent compound propofol rather than AziP*m-click* (**1**), but they yield affinities that are consistent with results from AziP*m-click* (**1**).

Second, our current investigation is limited to the employed biological system, a synaptosomal fraction derived from whole brain, and therefore, it is focused on synaptic proteins, including synaptic GABA_A_ receptors. Propofol has previously been shown to influence tonic receptor-mediated inhibition that is anticipated to contribute to observed hypnotic sedation and other anesthesia phenotypes, specifically amnesia (13, 14, 54). Because of the lack of extrasynaptic GABA_A_ receptor subunits within our proteome (data not shown), we could not determine the binding character for these receptors.

Finally, although the presented quantitative ABPP strategy allows the first platform for identifying alkylphenol protein targets relative to a native biological milieu, it does not directly identify the photoaffinity labeled residues. In part, this is a result of the challenging elution/digestion of labeled proteins and large modification size (>600 Da) of the biotin PEG3-conjugated AziP*m-click* (**1**) modification. Currently available cleavable biotin-*X*-azide linkers have variable cleavage efficiency and/or require reagents that perturb downstream quantitative labeling for native tissue-derived systems (55). Our efforts for non-quantifiable capture using cleaved biotin linker and AziP*m-click* (**1**) identified modifications on higher abundance proteins like the voltage-dependent cation channel (data not shown) that corresponded with earlier reports (56); however, residue-level modifications on lower abundance proteins, like the GABA_A_ receptor, remained undetected. Thus, we cannot confirm an interfacial location of sites in this study, although this location has been demonstrated in heterologous receptors (19). Future development of chemically active alkylphenol anesthetics, cleavable biotin linkers, as well as enhanced peptide enrichment and release methods may allow for increased capture efficiency permitting detection of modifications within the very low abundance photoaffinity labeled peptides.

##### Additional Alkylphenol-based Anesthetic Synaptic Targets

It is unlikely that a given drug will only bind and act on a single protein target within a proteome. In particular, the small general anesthetic molecules have been shown to bind to many different proteins (57). Although propofol is thought to have higher affinity for specific protein targets relative to volatile anesthetics, the projected affinities for major targets, as we observed with the GABA_A_ receptor, still remain in the low micromolar range. Therefore, it is not surprising that a number of targets (∼200) were captured due to the promiscuous binding associated with the general anesthetic. Whether the activity of every identified protein is altered upon alkylphenol binding is not clear and is not likely. However, some captured targets, in addition to the GABA_A_ receptor, have been reported as being influenced by propofol. Examples include syntaxin-1A (58), *N-*methyl-d-aspartate receptor (59), potassium/sodium hyperpolarization-activated cyclic nucleotide-gated channel 1 (60), as well as voltage-gated calcium channels (61) and potassium channels (62), all of which may contribute to desirable and/or undesirable pharmacological effects.

##### Concluding Remarks

Although the GABA_A_ receptor is considered to be an important target for general anesthetics, the mechanism of GABA_A_ receptor modulation remains unclear. In this investigation, we aimed to further understand alkylphenol binding to native receptors and to evaluate the molecular recognition elements that mediate affinity. Our results indicate that propofol binds to the assembled receptor in an asymmetric pattern, with greater affinity for β+/α− and α+/β− interfaces. Hydrogen bonding and cavity hydration were found to be the likely defining factors that contribute to the differential interfacial affinity and functional activity. In addition, this work suggests that the alkylphenol anesthetic proteome is large and complex, providing the opportunity to modulate activity at many targets. Finally, this work adds to current methodologies used for the identification of anesthetic targets and a better understanding of allosteric binding interactions.

## Experimental Procedures

### 

#### 

##### General Synthetic Procedures

Reagents and solvents were all used as acquired from commercial sources. ^1^H, ^13^C, and ^19^F NMR spectra were obtained on either a Bruker DMX 500 MHz or a Bruker DMX 360 MHz nuclear magnetic resonance spectrometer. The detailed synthetic methodology and associated NMR spectra for intermediates and AziP*m-click* (**1**) are provided in the supplemental material. Purity of AziP*m-click* (**1**) was determined using reverse phase-HPLC with a C-18 analytical column with a 60-min gradient from 40 to 70% acetonitrile in 0.1% formic acid at a 1 ml/min flow at ambient temperature (21–22 °C). AziP*m-click* (**1**) was monitored for UV-visible absorbance at 210 and 365 nm. The retention time for AziP*m-click* (**1**) was observed at 22.3 min with a purity of >96%.

##### Physicochemical Properties

The UV spectrum and extinction coefficient of the AziP*m-click* (**1**) diazirine absorption were obtained from known concentrations in methanolic solutions and gathered from the Varian Cary 300 Bio UV-visible spectrophotometer. Photoactivation of the diazirine was measured by the disappearance of the diazirine UV absorption peaks when exposed to 350 nm light (Rayonet RPR-3500 lamp) ∼6 cm from the light source. Maximum water solubility was approximated using the extinction coefficient. Calculated octanol/water partition coefficients were generated using XLOGP3 software 22 with default settings. The geometry-optimized structures for AziPm-*click*(**1**) was calculated at the B3LYP/6–311+G (2*d,p*) level of theory using Gaussian 09 (63).

##### Isothermal Titration Calorimetry

Isothermal titration calorimetry isotherms for binding to soluble protein model horse spleen apoferritin were conducted as reported previously (31) and were resolved using a VP-isothermal titration calorimetry microcalorimeter (MicroCal, Inc., Northampton, MA). Origin 5.0 software was used to best-fit thermodynamic parameters to the heat profiles.

##### 1-AMA Displacement Fluorescence Assay

1-AMA fluorescence inhibition has been reported as a reliable measurement of anesthetic occupation of the horse spleen apoferritin anesthetic site (64). 1-AMA displacement studies were conducted as described previously (29). The fluorescence intensity *versus* concentration data were fitted to variable slope Hill models to obtain the IC_50_ and Hill slope. The *K_D_* value was calculated using the Cheng-Prusoff equation to correct for the presence of the 1-AMA competitors.

##### Crude Synaptosome Preparation

Mouse crude synaptosomes were prepared as reported previously (65) with modifications. Male C57/B6 mice (8–12 weeks) were deeply anesthetized with isoflurane and intracardially perfused with ice-cold phosphate-buffered saline (PBS; pH 7.4) before decapitation. Brains were extracted and homogenized in ice-cold isolation buffer (IB; 0.32 m sucrose, 2.5 mm HEPES, 1 mm EDTA (pH 7.4)) (10% w/v%) in the presence of protease and phosphatase inhibitors. The homogenate was centrifuged at 1,000 × *g* for 10 min at 4 °C. The resulting supernatant was decanted, and the pellet was homogenized with an equal volume of IB and centrifuged at 1,000 × *g* for 10 min at 4 °C. Both supernatants were pooled and were centrifuged at 1,000 × *g* for 10 min at 4 °C. The supernatant was decanted and centrifuged at 12,000 × *g* for 20 min at 4 °C. The pellet was washed twice by resuspension of the pellet in 2× volumes of IB and centrifuged at 12,000 × *g* for 15 min at 4 °C. The resulting crude preparation of synaptosomes, now entirely free of the euthanizing isoflurane, was used in following experiments. All following protein contents are measured using BCA assay (Thermo Scientific). Animal care and experimental procedures involving mice were carried out according to a protocol approved by the IACUC of the University of Pennsylvania.

##### Synaptosomal Photoaffinity Labeling

Synaptosomes were resuspended to 1 mg of protein/ml in HEPES buffer medium (in mm: 140 NaCl, 5 KCl, 5 NaHCO_3_, 1.2 NaH_2_PO_4_, 1 MgCl_2_, 10 glucose, and 10 HEPES (pH 7.4)). Concentrations of AziP*m-click* (**1**) with or without the presence of concentrations of competitive ligands (propofol, ketamine, or fropofol) in dimethyl sulfoxide (DMSO) vehicle (<0.3% v/v) were added, and synaptosomes were gently vortexed for 10 s. The samples were allowed to equilibrate for 5 min before being transferred to a parafilm-sealed 1-mm path length quartz cuvette. The sample was then irradiated for 20 min at a peak bandwidth of 350 nm (Rayonet RPR-3500 lamp) ∼6 cm from the light source. Non-irradiated samples were left in the dark at ambient temperature (22–25 °C) for 20 min. All remaining procedures were conducted with restricted light exposure.

##### Fluorophore Conjugation for Proteome Detection

To 150 μg of photolabeled or control synaptosomes, 8 μl of 10% SDS in water and 2 μl of 1 mm dithiothreitol (DTT) in water were added. Samples were vortexed and heated at 65 °C for 10 min. After a brief cooling, final concentrations of 30 μm azide-PEG3-Fluor 488 (Click Chemistry Tools), 2 mm tris(3-hydroxypropyltriazolylmethyl)amine (Sigma), 1 mm ascorbic acid (Sigma), and 1 mm CuSO_4_·5H_2_O (Sigma) were added to each sample and vortexed vigorously. The samples were left in the dark for 1 h. After, 4× volume of chilled methanol, 1.5× of chilled chloroform, and 3× of chilled double distilled H_2_O were added and vortexed vigorously. Samples were centrifuged at 1,300 × *g* for 30 min, and both liquid layers were carefully removed. The protein pellet was washed with 500 μl of 1:1 (v/v) methanol/chloroform and centrifuged at 14,000 × *g* for 20 min at 4 °C. Washed pellets were air-dried for 10 min and resuspended in 25 μl of 1% SDS and 1% Triton X-100 in 50 mm Tris base buffer. An equal volume of 2× SDS Laemmli buffer was added, and 25 μg of protein was loaded without boiling to 4–15% SDS-polyacrylamide gel. Proteins were directly visualized within the gel using fluorescence and then stained with Coomassie G-250 stain. Fluorescent studies were normalized to Coomassie stain band intensity.

##### Heterologous Expression of GABA_A_ Receptor Subunits and Electrophysiological Recordings

GABA_A_ receptor expression in *X. laevis* oocytes was completed as described previously (40). cDNAs for GABA_A_ receptor α_1_, β_2_, and γ_2L_ subunits were generously provided by Dr. Robert Pearce (University of Wisconsin). All animal care and experimental procedures involving *X. laevis* frogs were carried out according to a protocol approved by the IACUC of Thomas Jefferson University. GABA_A_ receptor currents expressed in *X. laevis* oocytes were recorded as reported previously (40). Data acquisition and initial analysis were performed using pClamp 9.2/10.3 (Molecular Devices, Sunnyvale, CA). Macroscopic currents were low-pass filtered at 1 kHz and digitized at 2 kHz. Data were fit to a sigmoidal dose-response curve with variable Hill slope.

##### Hypnotic Activity and in Vivo Photolabeling in X. laevis Tadpoles

Behavioral activity was initially determined in albino *X. laevis* tadpoles (stages 45–47) as described previously (29, 33). All animal care and experimental procedures involving *X. laevis* tadpoles were carried out according to a protocol approved by the IACUC of the University of Pennsylvania.

##### Biotin Conjugation

To 750 μg of photolabeled or control synaptosome samples, 40 μl of 10% SDS and 2 μl of 5 mm DTT in water were added. Samples were then vortexed, heated for 10 min at 65 °C, and then briefly cooled. Final concentrations of 150 μm azide-biotin (Click Chemistry Tools), 2 mm tris(3-hydroxypropyltriazolylmethyl)amine (Sigma), 1 mm ascorbic acid (Sigma), and 1 mm CuSO_4_·5H_2_O (Sigma) were added to each sample and vortexed vigorously. The samples were left in the dark at ambient temperature (22–25 °C) for 1 h with mild agitation. Directly to each sample 4× volume chilled methanol, 1.5× chilled chloroform, and 3× chilled double distilled H_2_O were added. Samples were vortexed vigorously and centrifuged at 1,400 × *g* for 30 min at 4 °C. Both liquid layers were carefully removed, and the protein pellet was washed with 2 ml of 1:1 (v/v) chilled methanol/chloroform. Samples were centrifuged at 3,500 × *g* for 30 min at 4 °C. Protein pellets were briefly air-dried before further processing.

##### Sample Processing for ABPP Mass Spectrometry Studies

750 μg of biotin-conjugated protein sample was resuspended in 500 μl of 25 mm NH_4_HCO_3_ and 6 m urea in water. Next, 150 μl of 5% Triton X-100 in water, 50 μl of 10% SDS in water, and 1.5 μl of 0.5 m DTT were added. The samples were heated for 15 min at 65 °C. After briefly cooling, 14 μl of 0.5 m iodoacetamide in water was added, and the sample was left in the dark for 45 min. Insoluble debris was separated by centrifugation for 10 min at 14,000 × *g*. The supernatant was diluted to 4 ml with PBS, and 2 ml of PBS containing 100 μl of 50% streptavidin-agarose resin (Thermo Scientific) was added. Biotinylated proteins within the sample were captured over resin overnight at 4 °C with mild agitation. The resin was first washed with 6 ml of 1% SDS in PBS, and then 7 ml of 0.1 m urea in PBS followed by 10 ml of PBS. The resin underwent a final wash with 0.9 ml of 50 mm Tris-HCl and 1 mm CaCl_2_ in water (pH 8.0) and then resuspended in 200 μl of 50 mm Tris-HCl, 1 mm CaCl_2_ in water (pH 8.0), and 2 μg of porcine sequencing grade trypsin (Promega). Samples were digested overnight at 37 °C. Samples were then centrifuged at 2,000 × *g* for 4 min, and digest supernatant was decanted. Beads were washed in 100 μl of PBS centrifuged at 5,200 × *g* for 5 min, and the wash was combined with the digest supernatant. To the combined sample, trifluoroacetic acid (TFA) was added to 0.4% (v/v) or until pH <2. The sample was desalted with Oasis C18 10-mg columns (Waters) as described previously (66). The eluted sample was dried by speed vac and resuspended in 0.1 m HEPES buffer (pH 8.5). Samples were labeled with Tandem Mass Tag^TM^ 6-plex (TMTsixplex^TM^) (Thermo Scientific) with the UV(+) sample labeled with TMT^6^− 128 or 131 reagent, the propofol protection sample labeled with TMT^6^− 126 or 129 reagent, and the UV(−) sample labeled with TMT^6^− 127 or 130 reagent using product instructions. Appropriate corresponding TMTsixplex^TM^-labeled samples were pooled and dried by speed vac. The combined samples were resuspended in 0.5% acetic acid in water and pH-corrected with acetic acid until pH was <2. 40 μg of protein was desalted with C18 stage tips prepared in-house and dried by speed vac.

Samples were resuspended in 10 mm KH_2_PO_4_ (pH 2.6), 30% acetonitrile (v/v) in water, and fractionated by off-line strong cation exchange chromatography prior to mass spectrometry (MS) analysis similar to as reported previously (66). The full synaptosome proteome control was prepared similarly without TMTsixplex^TM^ labeling.

##### Mass Spectrometry Analysis

All TMT samples were analyzed with three-stage mass spectrometry (MS3) TMTsixplex^TM^ quantification workflow as described previously (34). Spectral analysis was conducted using Thermo Proteome Discoverer 2.0 (Thermo Scientific) and mouse non-redundant (gene-centric) FASTA database. Mascot searches allowed for variable oxidation of methionine (+15.9949 *m/z*) and static modifications of cysteine residues (+57.0215 *m/z*; iodoacetamide alkylation) and TMTsixplex^TM^ tags on lysine residues and peptide N termini (+229.162932 *m/z*). To establish the base synaptosomal proteome, searches allowed for variable oxidation of methionine (+15.9949 *m/z*) and static modifications of cysteine residues (+57.0215 *m/z*; iodoacetamide alkylation). All studies maintained trypsin enzyme specificity filtered with no greater than two missed cleavages. The MS2 spectral assignment was restricted to a specified false-positive rate of 1%, and a minimum of two unique peptides was required for protein identifications. Quantification was based on the theoretical *m/z* of the individual TMTsixplex^TM^ reporter ions as reported previously (34). Enrichment factor was defined as the mean (+)UV/(−)UV TMT ratio. Frequency distribution histograms of log2 values were generated using GraphPad Prism 7.0.

##### Western Blotting for Biotin-conjugated Protein Targets

750 μg of biotin-conjugated protein sample was resuspended via sonication in 500 μl of 25 mm NH_4_HCO_3_ and 6 m urea in water. Following that, 150 μl of 5% Triton X-100 in water, 50 μl of 10% SDS in water, and 1.5 μl of 0.5 m DTT were added. The samples were heated for 15 min at 65 °C. Insoluble debris was separated by centrifugation for 10 min at 14,000 × *g*. The supernatant was diluted to 1 ml with PBS, and 50 μl was removed for the input sample. An additional 5 ml of PBS containing 100 μl of 50% streptavidin-agarose resin (Thermo Scientific) was added. Biotinylated proteins were captured over resin overnight at 4 °C with mild agitation. The resin was first washed with 6 ml of 1% SDS in PBS and then 7 ml of 0.1 m urea in PBS followed by 10 ml of PBS. The resin underwent a final wash with 0.9 ml of PBS and then was resuspended in 100 μl of 2× SDS Laemmli buffer containing 100 mm DTT. Samples were then incubated with agitation at 37 °C for 30 min, centrifuged at 700 × *g* for 2 min, and heated for 15 min at 90 °C. 50 μl of 2× SDS Laemmli buffer containing 100 mm DTT was joined to the input sample and heated for 5 min at 90 °C. Samples were centrifuged at 14,000 × *g* for 10 min prior to electrophoresis using 4–15% SDS-polyacrylamide gels with 10 μl of each sample introduced into each well. Proteins were then transferred to PVDF membranes. The membranes were blocked for 1 h with 2.5% BSA in Tris-buffered saline containing 0.1% Tween 20 (v/v; TBST). Membranes were incubated with GABA_A_ receptor subunit antibodies overnight at 4 °C. All antibodies for GABA_A_ receptor subunits were purchased from Santa Cruz Biotechnology, Inc., and included rabbit or goat polyclonal α1 ((A-20) sc-31405), α3 ((J-23) sc-122603), β1((N-19) sc-7361), β2 ((C-20) sc-7362), and γ2 ((Q-18) sc-101963) antibodies and monoclonal β3 ((D-12) sc-376252) antibody. For GABA_A_ receptor subunit analysis, membranes were washed three times with TBST prior to a 2-h incubation with appropriate HRP-conjugated secondary antibody at room temperature. All membranes were then washed twice with TBST and once with Tris-buffered saline (TBS) before being developed with Amersham Biosciences ECL select reagent and scanned. Only the net ratio of intensity-detected band(s) between 75 and 50 kDa was considered. The elution intensities were normalized to the corresponding input sample. Samples showing no detectable band elution were set to a net ratio of intensity of 0. Studies were conducted in quadruplicate and are represented as the fraction of the corresponding input.

##### Molecular Dynamics Simulations

A model of the α_1_β_3_γ_2_ GABA_A_ receptor was built by mutating 31 residues in the β subunits from an α_1_β_1_γ_2_ GABA_A_ model 3 reported in Hénin *et al.* (35). The mutations were made using the MUTATOR plugin of VMD (67). AutoDock Vina (68) was used to generate initial coordinates for propofol; default parameters were used, and the search space included the entire pentamer. AutoDock Vina returned at least one pose for each subunit interface; the ligand conformation with the best score was chosen for each site. The complex (GABA_A_ receptor and five propofol molecules) was then placed in a 109 × 109 Å phosphatidylcholine membrane aligned parallel to the *xy* plane using CHARMM-GUI membrane builder (69). The system was solvated to a total height in *z* of 139 Å, followed by the addition of sodium and potassium ions that neutralized the system and brought the salt concentration to 0.15 m. The complete simulation system contained about 167,000 atoms.

The CHARMM36 force field was used for protein (70, 71) and phospholipid (72) parameters, with parameters for TIP3P waters (73) and ions (74) corresponding to those traditionally used with CHARMM-based force fields. Propofol parameters relied on atom types from CHARMM36, as described in LeBard *et al.* (75), further parameterization and use of a CMAP potential was required to accurately enforce coupling between rotation of the hydroxyl and isopropyl groups due to steric clashes.

Atomistic molecular dynamics simulations were run with NAMD version 2.10 (76). All simulations used periodic boundary conditions and particle mesh Ewald (PME) electrostatics. Interactions between non-bonded atoms were cut off at 12 Å, and bonds involving hydrogen were constrained using the SHAKE/RATTLE algorithm. A Langevin thermostat and barostat were used to maintain a temperature and pressure of 300 K and 1 atm, respectively, and vanishing surface tension was imposed. The simulation time step was 2 fs. Following the system generation, 30,000 minimization steps and a 7-ns equilibration protocol that gradually softened restraints on the protein and ligand were run. Subsequently, we ran a 200-ns production run with soft harmonic restraints on the Cα atoms (*k* = 0.5 kcal/mol/Å^2^). The probability of hydrogen bonding was calculated using a VMD script that measured the fraction of frames in which propofol was hydrogen bonding to any residue in the site, detected using the VMD geometric criterion with a distance cutoff of 3.3 Å and an angle cutoff of 40°. The first 50 ns of the production run were not included in the analysis.

Binding affinities were calculated using the AFEP method, a theoretically exact method that involves gradually decoupling (reducing interaction strength) the ligand and the binding site throughout an MD simulation (44, 77). The decoupling free energy was then corrected by the ligand solvation free energy, as well as the entropic cost of transferring the ligand from the available volume per molecule in the standard state (1,660 Å^3^) to the volume of the ligand-binding site, yielding the standard Gibbs free energy of binding, Δ*G*^0^. The dissociation constant *K_D_* was calculated using the relationship *K_D_* = exp(−Δ*G*^0^/*RT*). Implementation of the method was very closely based on the procedure used by LeBard *et al.* (75) for propofol binding to intrasubunit site transmembrane domains of *Gloeobacter* ligand-gated ion channel. Decoupling of propofol from each of four interfaces was carried out in four separate simulations, over 24 windows, with 1 ns/window for a total of 24 ns per interfacial binding site.

The probability of propofol hydrogen bond formation (*P*_hb_) was estimated by calculating the frequency that a single hydrogen bond with the propofol hydroxyl was detected over the course of the equilibrium MD simulation. Molecular images in Figs. 6, *A* and *C–E,* and 7 were generated using VMD (67), and the data in Fig. 6*B* was plotted and fit using python scripts.

##### Statistics

GraphPad Prism 7.0, ChemDraw Professional 15.0, and Microsoft Excel, unless otherwise noted, were used for figure preparation and statistical data analysis.

##### Supplemental Material

^1^H, ^13^C, and ^19^F NMR spectra and detailed synthetic methods of AziP*m-click* (**1)** are presented in supplemental S2–S32. The identified propofol-specific proteome is presented in supplemental Table 1.

## Author Contributions

K. A. W., X. W., B. A. G., J. H., G. B., S. M., R. S., R. G. E., M. C., and W. P. D. participated in research design. K. A. W., J. H., B. J. P., X. W., S. M., W. P. D., and R. S. conducted the experiments. X. W., B. A. G., G. B., R. S., S. M., J. H., W. P. D., and B. J. P. contributed new reagents or analytic tools. K. A. W., X. W., G. B., S. M., J. H., W. P. D., and R. S. performed data analysis. K. A. W., X. W., B. A. G., J. H., G. B., S. M., R. G. E., M. C., and W. P. D. wrote or contributed to the writing of the manuscript.

## Supplementary Material

Supplemental Data

## Footnotes

2 The abbreviations used are:

propofol: 2,6-diisopropylphenol
ABPP: affinity-based protein profiling
MD: molecular dynamics
AFEP: alchemical free energy perturbation
1-AMA: 1-aminoanthracene
MS: mass spectrometry
TMT: tandem mass tag
fropofol: 2-fluoro-1,3-diisopropylbenzene
CI: confidence interval.
