# Supplementary material for: A Novel Bifunctional Alkylphenol Anesthetic Allows Characterization of γ-Aminobutyric Acid, Type A (GABAA), Receptor Subunit Binding Selectivity in Synaptosomes
Source: J Biol Chem. 2016 Jul 26;291(39):20473–86. doi: 10.1074/jbc.M116.736975 (PMC5034043; doi:10.1074/jbc.M116.736975)
Supplement: Supplemental Data [file supp_291_39_20473__index.html]

A Novel Bifunctional Alkylphenol Anesthetic Allows Characterization of GABAA Receptor Subunit Binding Selectivity in Synaptosomes — A Novel Bifunctional Alkylphenol Anesthetic Allows Characterization of γ-Aminobutyric Acid, Type A (GABAA), Receptor Subunit Binding Selectivity in Synaptosomes — Propofol-binding Sites in Native Synaptic GABAA Receptor — Supplemental Data 

# A Novel Bifunctional Alkylphenol Anesthetic Allows Characterization of γ-Aminobutyric Acid, Type A (GABAA), Receptor Subunit Binding Selectivity in Synaptosomes

## Supplemental Data

- Supplemental Information S2-32 (.pdf, 1.6 MB) - Supplemental Information S2-32
- Supplemental Table 1 (.xls, 90 KB) - Supplemental Table 1
