# Supplementary material for: A Novel Bifunctional Alkylphenol Anesthetic Allows Characterization of γ-Aminobutyric Acid, Type A (GABAA), Receptor Subunit Binding Selectivity in Synaptosomes
Source: J Biol Chem. 2016 Jul 26;291(39):20473–86. doi: 10.1074/jbc.M116.736975 (PMC5034043; doi:10.1074/jbc.M116.736975)

SUPPLEMENTARY INFORMATION

A novel bifunctional alkylphenol anesthetic allows characterization of $GABA_A$ receptor subunit binding selectivity in synaptosomes.

Kellie A. Woll, Sruthi Murlidaran, Benika J. Pinch, Jérôme Hénin, Xiaoshi Wang, Reza Salari, Manuel L. Covarrubias, William P. Dailey, Grace Brannigan, Benjamin A. Garcia, Roderic G. Eckenhoff

TABLE OF CONTENTS

Page	
S-2 - S-7	SYNTHETIC METHODS
S-8 - S32	^{1}H, ^{13}C AND ^{19}F NMR SPECTRA

SYNTHETIC METHODS

Synthesis of 2-((prop-2-yn-1-yloxy)methyl)-5-(3-(trifluoromethyl)-3*H*-diazirin-3-yl)phenol (AziP*m-click* (1))

2,2,2-Trifluoro-1-(3-(methoxymethoxy)-4-methylphenyl)ethan-1-one (3): Under an argon atmosphere, a solution of **2** (5.0 g; 21.6 mmol) in dry THF (23 mL) and magnesium metal (0.57 g; 23.5 mmol) were added to a 50 mL rbf equipped with a condenser and stir bar. The flask was heated slowly to initiate the reaction, and then was allowed to react without external heating. Once the exothermic reaction was finished, the contents were heated to reflux for 20 minutes to ensure complete consumption of **2**. The flask was cooled in an ice-salt bath for 25 minutes, causing a white precipitate to form. The condenser was replaced with an addition funnel containing 2,2,2-trifluoro-1-pyrollidin-1-ylethanone (2.9 g; 17.4 mmol) in dry THF (4.3 mL) and the amide solution was added dropwise over 30 minutes at 0 °C while stirring. After the addition, the mixture was stirred in the ice bath for 1 hour. The reaction was then quenched with saturated aqueous NH_4Cl solution (6 mL), and the mixture was vacuum-filtered. The resulting liquid was dried over $MgSO_4$, and the solvent was removed under reduced pressure to give 5.4 g of a yellow liquid. Distillation under high vacuum yielded 3.2 g (72%) of **3** as a yellow liquid, bp 59°C (35 mTorr). ^{1}H NMR (500 MHz, $CDCl_3$): δ 7.74 (1 H, s), 7.61 (1 H, d, J = 8 Hz), 7.29 (1 H, d, J = 8 Hz), 5.27 (2 H, s), 3.50 (3 H, s), 2.34 (3 H, s). ^{13}C NMR (125 MHz, $CDCl_3$): δ 179.89 (q, $J_{C\text{-}F}$= 34 Hz), 155.80, 136.95, 131.15, 128.90, 123.81, 116.79 (q, $J_{C\text{-}F}$= 290 Hz), 114.14, 94.43, 56.10, 16.71 ppm. ^{19}F NMR (340 MHz, $CDCl_3$) : δ -71.2 ppm. HRMS *m/z* calcd for $C_{11}H_{12}F_3O_3$ (M + H^+) 249.0738; found 249.0742.

2,2,2-Trifluoro-1-(3-(methoxymethoxy)-4-methylphenyl)ethan-1-one oxime (4): To a 25 mL rbf with stir bar was added **3** (1.0 g; 4 mmol), hydroxylamine hydrochloride (0.35 g; 4 mmol), and pyridine (10 mL). The flask was heated in an oil bath at 60 °C for 4 hours. The mixture was evaporated to remove volatiles and the residue was partitioned between methylene chloride (25 mL) and water (25 mL), and the separated organic layer was washed with additional water (20 mL). The organic solution was dried (Na_2SO_4) and was evaporated *in vacuo* to give 1.0 g of yellow oil. Crystallization from hexanes produced 600 mg (58%) of **4** as colorless needles, mp. 101-102 °C. ^{1}H NMR (500 MHz, $CDCl_3$): δ 9.19 (1 H, s), 7.26 (2 H, d, J = 8 Hz), 7.09 (1 H, d, J = 8 Hz), 5.25 (2 H, s), 3.52 (3 H, s), 2.31 (3 H, s). ^{13}C NMR (125 MHz, $CDCl_3$): δ 155.28, 147.44 (q, $J_{C\text{-}F}$= 33 Hz), 130.90, 130.62, 124.41, 121.80, 120.64 (q, $J_{C\text{-}F}$= 274 Hz), 114.18, 94.61, 56.18, 16.37 ppm. ^{19}F NMR (340MHz, $CDCl_3$): δ - 66.6 ppm. HRMS *m/z* calcd for $C_{11}H_{11}F_3NO_3$ $(M\text{-}H)^-$ 262.0691; found 262.0690.

2,2,2-Trifluoro-1-(3-(methoxymethoxy)-4-methylphenyl)ethan-1-one O-tosyl oxime (5): In a 25 mL rbf with stir bar, **4** (280 mg; 1.06 mmol) was dissolved in methylene chloride (7.3 mL). While stirring, 4-(N,N-dimethylamino)pyridine (6.2 mg; 0.05 mmol), *p*-toluenesulfonyl chloride (0.21 g; 1.16 mmol), and triethylamine (0.15 g; 208 µL; 1.5 mmol) were added to the flask. The mixture was stirred for 24 hours under nitrogen atmosphere at room temperature. Water (20 mL) and methylene chloride (20 mL) were then added to the reaction mixture, and the separated organic phase was washed with additional water (20 mL). The mixture was concentrated *in vacuo* to give 440 mg of a pale yellow crystalline solid. The crude product was purified by column chromatography with silica gel using 8% EtOAc/hexanes to give 360 mg (81%) of **7** as a clear crystalline

solid, mp 65-66 °C. ^{1}H NMR (500 MHz, $CDCl_3$): δ 7.89 (2 H, d, J = 8.5 Hz), 7.38 (2 H, d, J = 8.5 Hz), 7.23 (1 H, d, J = 8 Hz), 7.11 (1 H, s), 6.96 (1 H, d, J = 8 Hz), 5.20 (2 H, s), 3.50 (3 H, s), 2.49 (3 H, s), 2.28 (3 H, s). ^{13}C NMR (125 MHz, $CDCl_3$): δ 155.46, 153.9 (q, $J_{C\text{-}F}$ = 38 Hz), 146.06, 131.90, 131.34, 131.04, 129.88, 129.26, 122.88, 121.65, 120.78, 118.57, 116.58 (q, $J_{C\text{-}F}$ = 56.6 Hz), 113.82, 94.66, 56.12, 21.77, 16.56 ppm. ^{19}F NMR (340 MHz, $CDCl_3$): δ - 66.6 ppm. HRMS calcd for $C_{18}H_{19}F_3NO_5S$ $(M+H)^+$ 418.0936; found 418.0928.

3-(3-(Methoxymethoxy)-4-methylphenyl)-3-(trifluoromethyl)diaziridine (6): To a solution of **5** (340 mg; 0.82 mmol) in diethyl ether (2 mL) in a 50 mL rbf equipped with stir bar and dry ice gas condenser was added excess liquid ammonia at -78 °C. The mixture was stirred overnight and allowed to warm to room temperature. The remaining residue was partitioned between diethyl ether (35 mL) and water (40 mL). The organic layer was washed with additional water (20 mL). The ether solution was dried over Na_2SO_4, and the solvent was removed to give 215 mg (100%) of **6** as a white solid, mp 79-80 °C. ^{1}H NMR (500 MHz, $CDCl_3$): δ 7.29 (1 H, s), 7.20-7.15 (2 H, m), 5.23 (1 H, d, J = 7 Hz), 5.20 (1 H, d, J = 7 Hz), 3.50 (3 H, s), 2.79 (1 H, d, J = 9 Hz), 2.26 (3 H, s), 2.24 (1 H, d, J = 9 Hz). ^{13}C NMR (125 MHz, $CDCl_3$): δ 155.50, 130.99, 130.38, 129.78, 123.56 (q, $J_{C\text{-}F}$= 278 Hz), 121.21, 113.35, 94.56, 57.93 (q, $J_{C\text{-}F}$= 35 Hz), 56.13, 16.19 ppm. ^{19}F NMR (340 MHz, $CDCl_3$): δ -75.5 ppm. HRMS *m/z* calcd for $C_{11}H_{14}F_3N_2O_2$ $(M+H)^+$ 263.1007; found 263.1010.

3-(3-(Methoxymethoxy)-4-methylphenyl)-3-(trifluoromethyl)-3*H*-diazirine (7): To a 50 mL rbf with stir bar was added **6** (1.0 g; 3.82 mmol), PDC (2.0 g; 5.32 mmol), and methylene chloride (10 mL). The mixture was stirred overnight at room temperature. The

solution was diluted with hexanes (10 mL) and flushed through a short plug of silica gel with more hexanes. Evaporation of volatiles left 0.89 g (89%) of **7** as a clear light yellow liquid. ^{1}H NMR (500 MHz, $CDCl_3$): δ 7.17 (1 H, d, J = 8.5 Hz), 6.85 (1 H, s), 6.80 (1 H, d, J = 8.5 Hz), 5.19 (2 H, s), 3.50 (3 H, s), 2.25 (3 H, s). ^{13}C NMR (125 MHz, $CDCl_3$): δ 155.69, 131.13, 129.59, 127.74, 122.20 (q, $J_{C\text{-}F}$= 274 Hz), 119.85, 111.83, 94.56, 56.08, 28.45 (q, $J_{C\text{-}F}$= 40 Hz), 16.09 ppm. ^{19}F NMR (340 MHz, $CDCl_3$): δ -65.3 ppm. HRMS *m/z* calcd for $C_{11}H_{10}F_3N_2O_2$ $(M\text{-}H)^-$ 259.0694; found 259.0695.

3-(4-(Bromomethyl)-3-(methoxymethoxy)phenyl)-3-(trifluoromethyl)-3*H*-diazirine (8): In a 10 mL rbf with stir bar, **7** (0.47 g; 1.8 mmol), NBS (0.32 g; 1.8 mmol), and CCl_4 (3.5 mL) were combined. The flask was equipped with a condenser and a nitrogen balloon and placed in an oil bath preheated to 90 °C. Incandescent light was shone directly on the flask. When the reaction was complete as monitored by TLC, the product was dissolved in hexanes (5 mL) and flushed through a pipette containing Celite (2 cc). The resulting mixture was evaporated *in vacuo* to give 0.63 g of a yellow liquid. The product was then flushed through a plug of silica gel (15 cc) with hexanes, and the solvent was evaporated to yield 0.5 g (83%) of **8** as a colorless liquid. ^{1}H NMR (500 MHz, $CDCl_3$): δ 7.37 (1 H, d, J = 8 Hz), 6.88-6.84 (2 H, m), 5.27 (2 H, s), 4.53 (2 H, s), 3.52 (3 H, s). ^{13}C NMR (125 MHz, $CDCl_3$): δ 155.25, 151.46, 131.22, 131.14, 131.05, 128.65, 121.99 (q, $J_{C\text{-}F}$= 275 Hz), 120.00, 112.28, 94.74, 94.59, 56.59, 56.44, 56.10, 33.98, 28.42 (q, $J_{C\text{-}F}$= 40 Hz), 27.48, 16.11 ppm. ^{19}F NMR (340 MHz, $CDCl_3$): δ -65.1 ppm. HRMS *m/z* calcd for $C_{11}H_9BrF_3N_2O_2$ $(M\text{-}H)^+$ 336.9799; found 336.9799.

3-(3-(Methoxymethoxy)-4-((prop-2-yn-1-yloxy)methyl)phenyl)-3-(trifluoromethyl)-3*H*-diazirine (9): To a 10 mL rbf with stir bar was added 60% dispersion of sodium

hydride in mineral oil (0.06 g; 1.50 mmol) under nitrogen. The oil was removed by washing with hexanes (5 mL). Dry THF (2 mL) was then added, forming a cloudy white suspension. The mixture was cooled in an ice-water bath to 0 °C. To this mixture was added propargyl alcohol (0.08g; 79µL; 1.37 mmol) via syringe which caused bubbling. After the bubbling stopped, a solution of **8** (0.31 g; 0.91 mmol) dissolved in THF (1 mL) was added. The reaction was stirred at room temperature overnight. The mixture was dissolved in water (15 mL) and was extracted with diethyl ether (3 x 15 mL). The combined organic layers were washed with water (3 x 15 mL), and then dried over Na_2SO_4. The mixture was evaporated *in vacuo* to give 0.22 g (77%) of a yellow oil. The product was purified by column chromatography using silica gel and 5% ethyl acetate/hexanes, giving 0.13 g (45%) of **9** as a colorless oil, R_f = 0.33 (5% EtOAc/hexanes). ^{1}H NMR (500 MHz, $CDCl_3$): δ 7.43 (1 H, d, J = 8.5 Hz), 6.89 (1 H, d, J = 8.5 Hz), 6.88 (1H, s), 5.20 (2 H, s), 4.66 (2 H, s), 4.22 (2 H, d, J = 2.5 Hz), 3.48 (3 H, s), 2.46 (1 H, t, J = 2.5 Hz). ^{13}C NMR (125 MHz, $CDCl_3$): δ 154.96, 129.73, 129.35, 128.73, 122.09 (q, J_{C-F}= 275 Hz), 119.99, 111.94, 79.58, 74.66, 66.06, 57.74, 56.27, 28.45 (q, J_{C-F}= 40 Hz) ppm. ^{19}F NMR (340 MHz, CDCl3): δ -65.3 ppm. HRMS *m/z* calcd for $C_{14}H_{12}F_3N_2O_3$ $(M-H)^-$ 313.0800; found 313.0812.

2-((Prop-2-yn-1-yloxy)methyl)-5-(3-(trifluoromethyl)-3*H*-diazirin-3-yl)phenol (AziPm-click, 1). In a 10 mL rbf with stir bar, **9** (65 mg; 0.21 mmol) was dissolved in methylene chloride (2.3 mL) at room temperature. While stirring, activated (hot) $NaHSO_4$•SiO_2 (43 mg) was added. After 4 hours the reaction was complete, as shown by TLC, and the mixture was dissolved in methylene chloride (2 mL) and run through a pipette containing silica gel (2 cc). The resulting solution was evaporated *in vacuo* and

then evaporated under high vacuum to give 37.3 mg of a clear oil. The product was purified by column chromatography with silica gel using 15% EtOAc/hexanes to give 28.5 mg (52%) of **12**, a colorless oil. $R_f = 0.3$ (15% EtOAc/hexanes). ^{1}H NMR (500 MHz, $CDCl_3$): δ 7.17 (1 H, s), 7.09 (1 H, d, J = 8 Hz), 6.73 (1 H, s), 6.66 (1 H, d, J = 8 Hz), 4.80 (2 H, s), 4.25 (2 H, d, J = 2 Hz), 2.54 (1 H, t, J = 2 Hz). ^{13}C NMR (125 MHz, $CDCl_3$): δ 156.50, 131.12, 129.22, 123.33, 122.21 (1, $J_{C\text{-}F}$= 274 Hz), 118.18, 115.04, 78.19, 76.39, 70.30, 57.94, 28.44 (q, $J_{C\text{-}F}$= 40 Hz) ppm. ^{19}F NMR (340 MHz, $CDCl_3$): δ -65.3 ppm. HRMS *m/z* calcd for $C_{12}H_9F_3N_2O_2$ $(M)^+$ 270.0616; found 270.0618.

^{1}H, ^{13}C AND ^{19}F NMR SPECTRA

TABLE OF CONTENTS

Page

S-9 ^{1}H NMR spectrum (500 MHz) of **3**

S-10 ^{13}C NMR spectrum (125 MHz) of **3**

S-11 ^{19}F NMR spectrum (340 MHz) of **3**

S-12 ^{1}H NMR spectrum (500 MHz) of **4**

S-13 ^{13}C NMR spectrum (125 MHz) of **4**

S-14 ^{19}F NMR spectrum (340 MHz) of **4**

S-15 ^{1}H NMR spectrum (500 MHz) of **5**

S-16 ^{13}C NMR spectrum (125 MHz) of **5**

S-17 ^{19}F NMR spectrum (340 MHz) of **5**

S-18 ^{1}H NMR spectrum (500 MHz) of **6**

S-19 ^{13}C NMR spectrum (125 MHz) of **6**

S-20 ^{19}F NMR spectrum (340 MHz) of **6**

S-21 ^{1}H NMR spectrum (500 MHz) of **7**

S-22 ^{13}C NMR spectrum (125 MHz) of **7**

S-23 ^{19}F NMR spectrum (125 MHz) of **7**

S-24 ^{1}H NMR spectrum (500 MHz) of **8**

S-25 ^{13}C NMR spectrum (340 MHz) of **8**

S-26 ^{19}F NMR spectrum (125 MHz) of **8**

S-27 ^{1}H NMR spectrum (500 MHz) of **9**

S-28 ^{13}C NMR spectrum (340 MHz) of **9**

S-29 ^{19}F NMR spectrum (125 MHz) of **9**

S-30 ^{1}H NMR spectrum (500 MHz) of **1**

S-31 ^{13}C NMR spectrum (340 MHz) of **1**

S-32 ^{19}F NMR spectrum (125 MHz) of **1**

^{1}H NMR (500 MHz, $CDCl_3$) 2,2,2-trifluoro-1-(3-(methoxymethoxy)-4-methylphenyl)ethan-1-one (3)

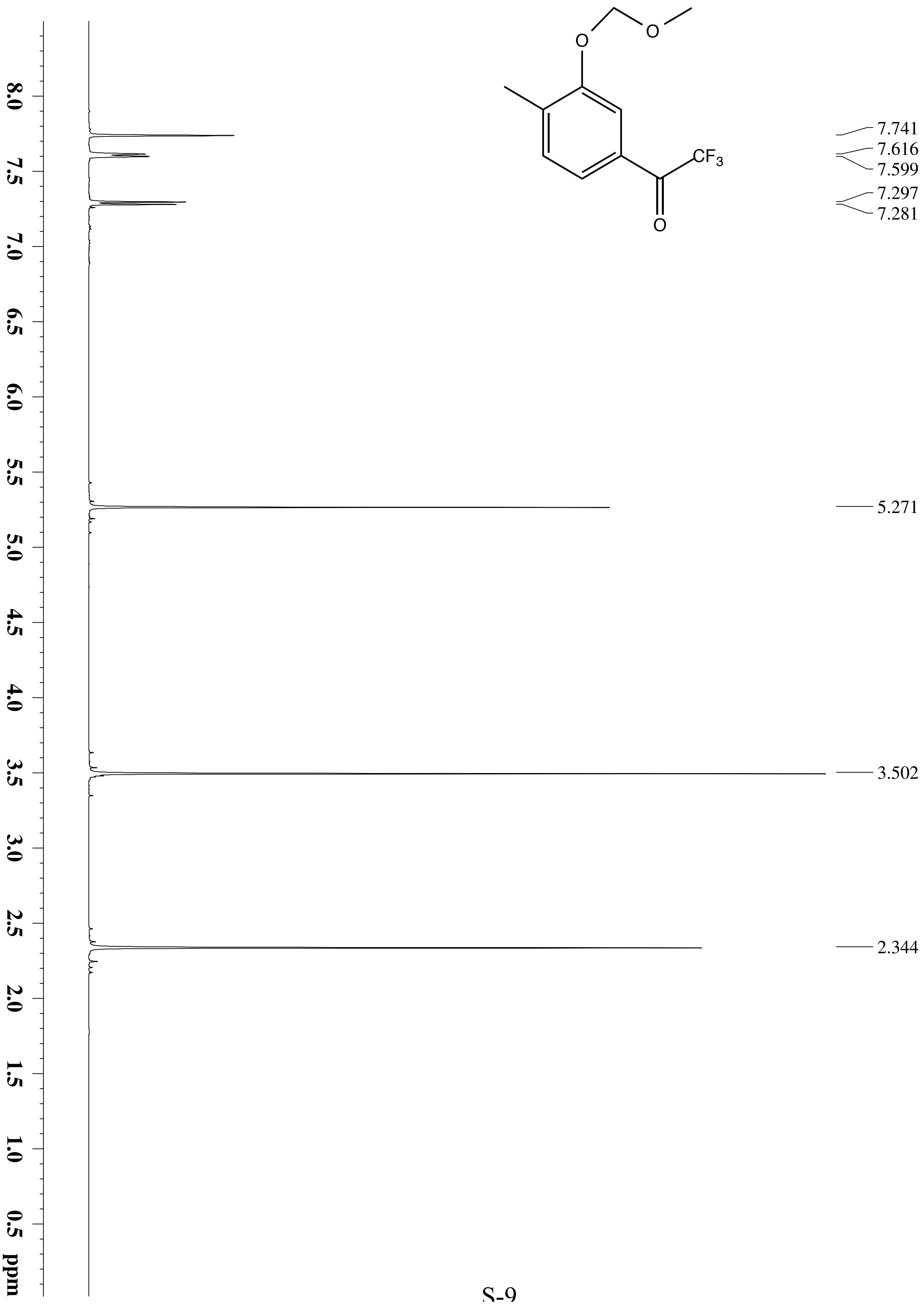

^{13}C NMR (500 MHz, CDCl3) 2,2,2-trifluoro-1-(3-(methoxymethoxy)-4-methylphenyl)ethan-1-one (3)

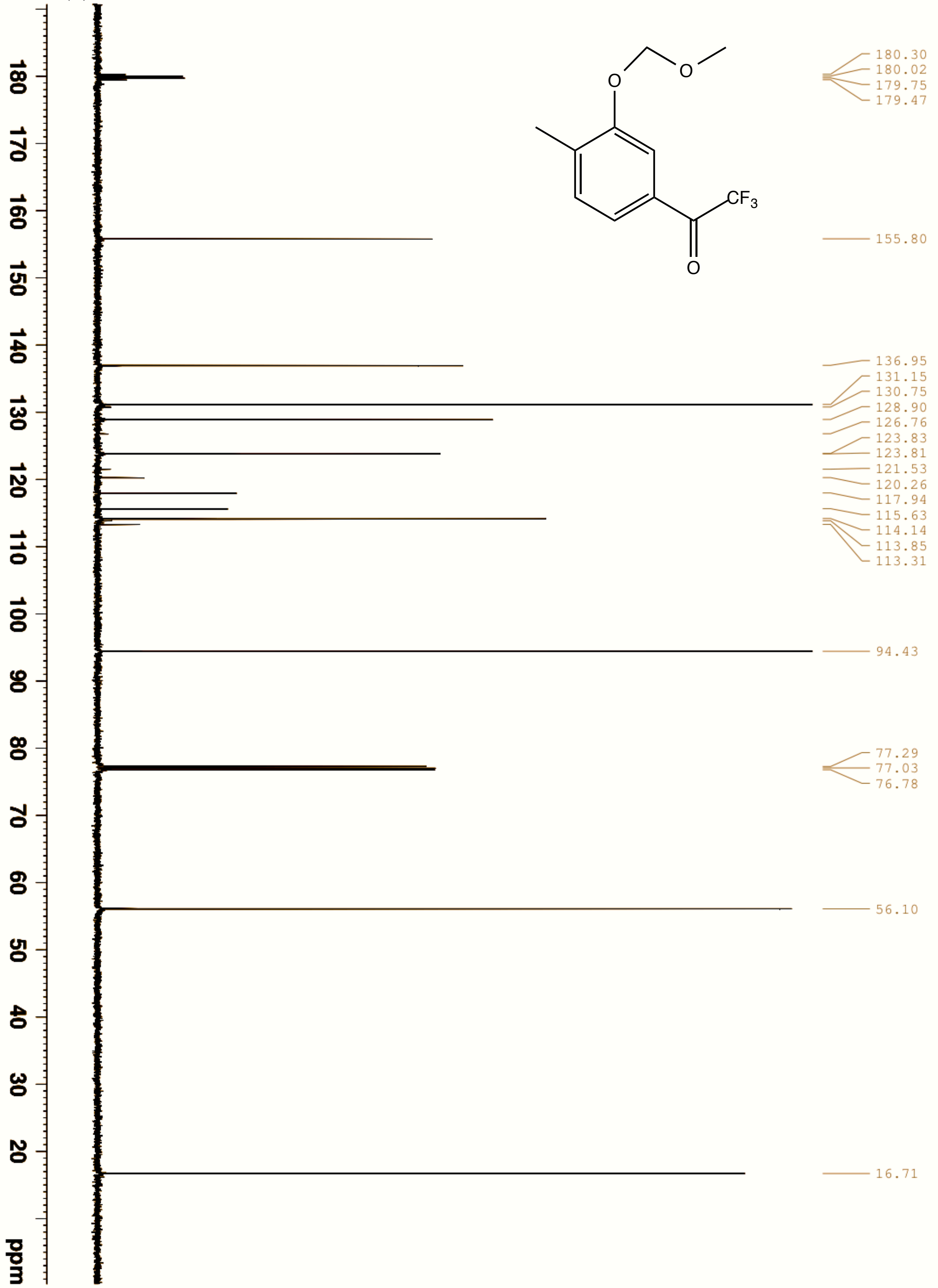

^{19}F NMR (360 MHz, $CDCl_3$) 2,2,2-trifluoro-1-(3-(methoxymethoxy)-4-methylphenyl)ethan-1-one (3)

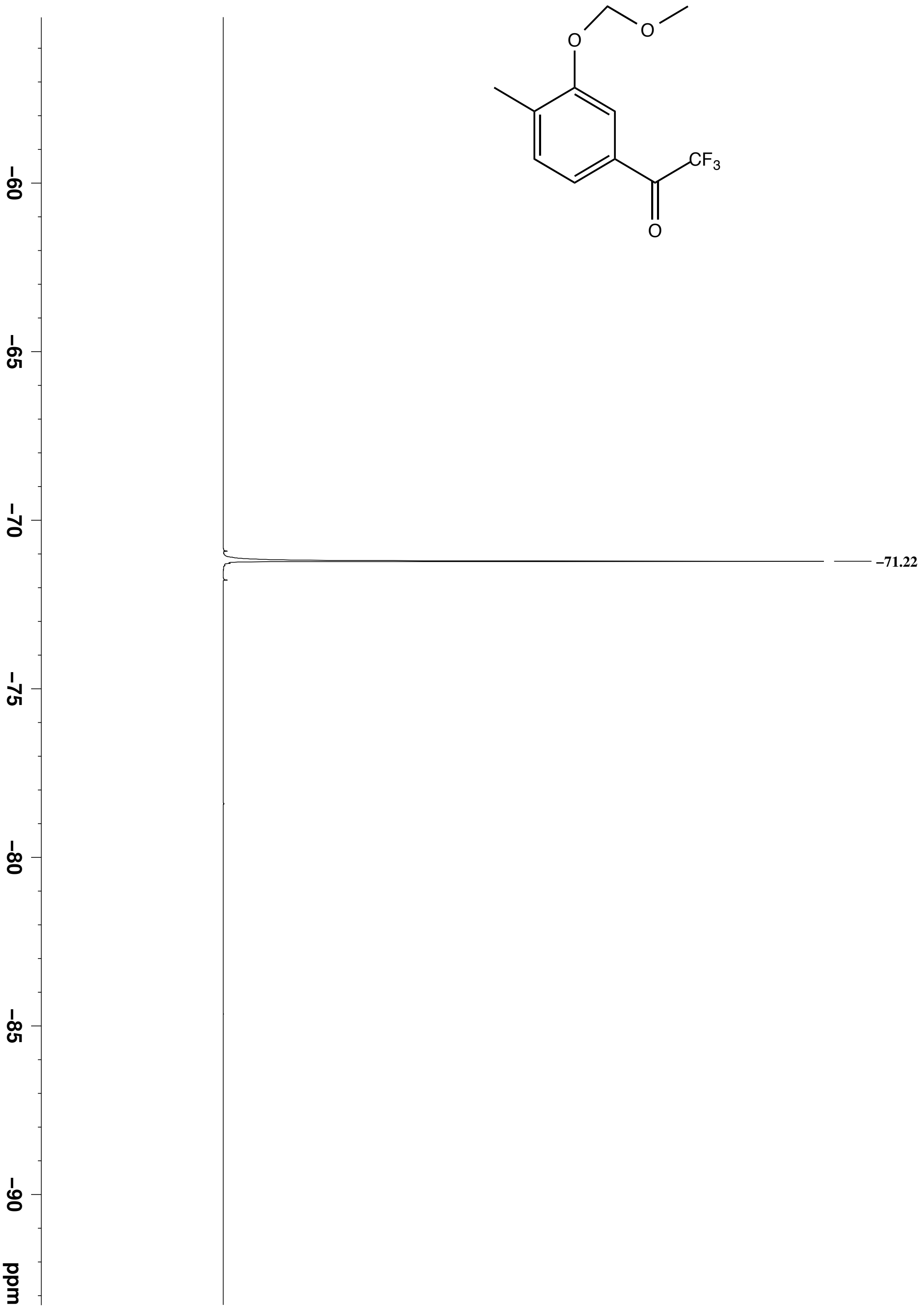

^{1}H NMR (500 MHz,CDCl$_3$) 2,2,2-trifluoro-1-(3-(methoxymethoxy)-4-methylphenyl) ethan-1-one oxime (4)

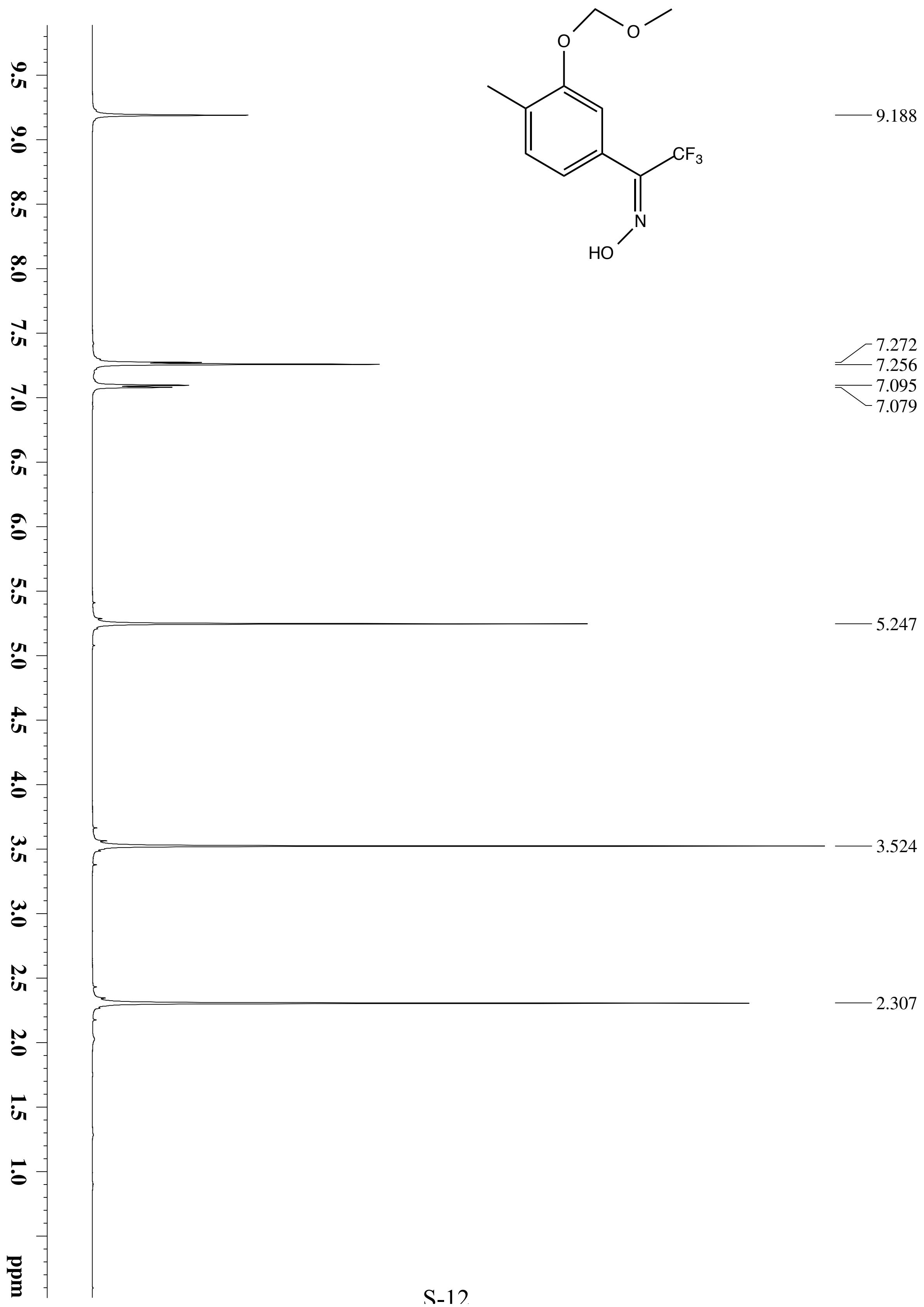

^{13}C NMR (500 MHz, $CDCl_3$) 2,2,2-trifluoro-1-(3-(methoxymethoxy)-4-methylphenyl) ethan-1-one oxime (4)

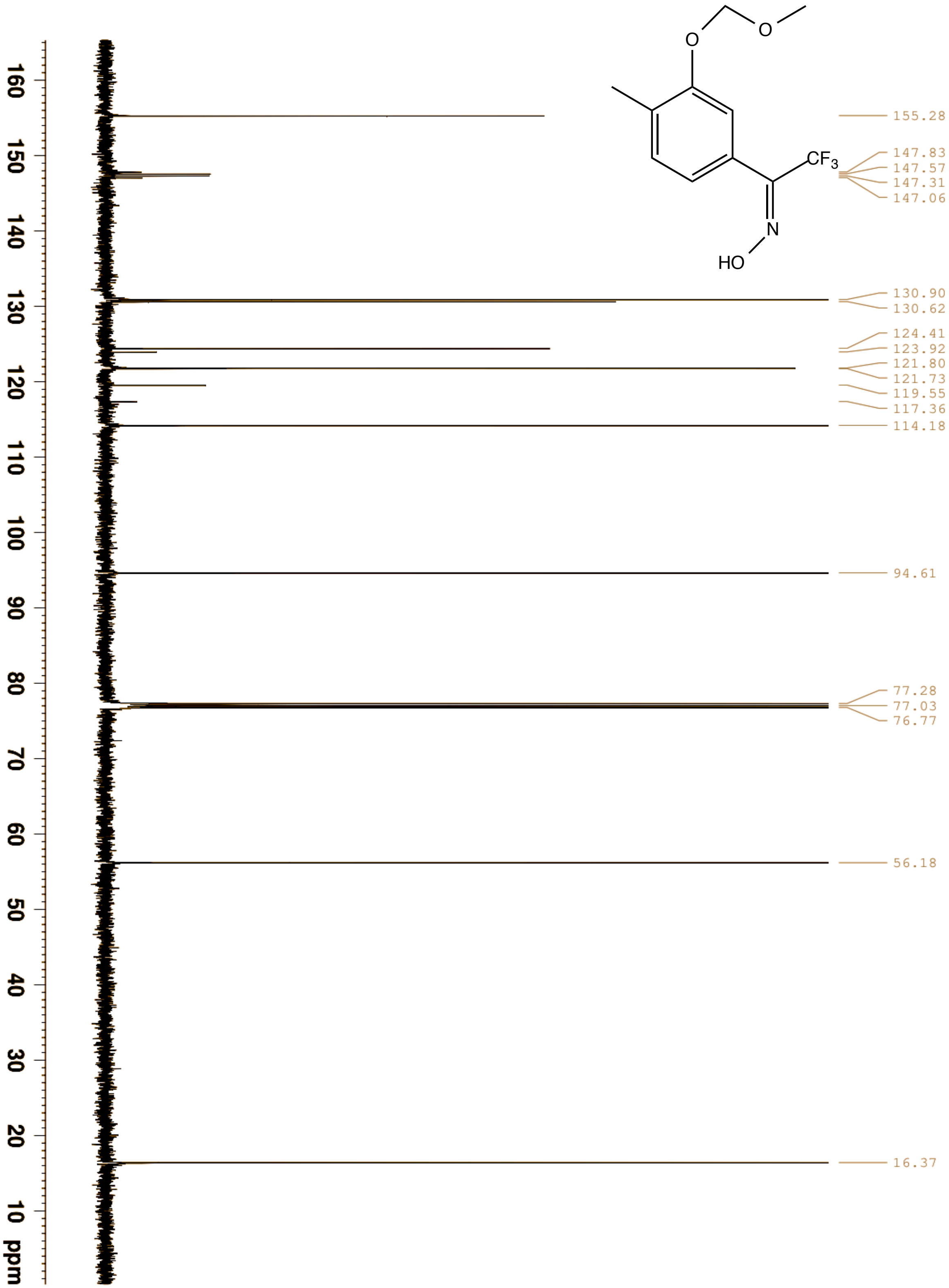

^{19}F NMR (360 MHz, $CDCl_3$) 2,2,2-trifluoro-1-(3-(methoxymethoxy)-4-methylphenyl)ethan-1-one oxime (4)

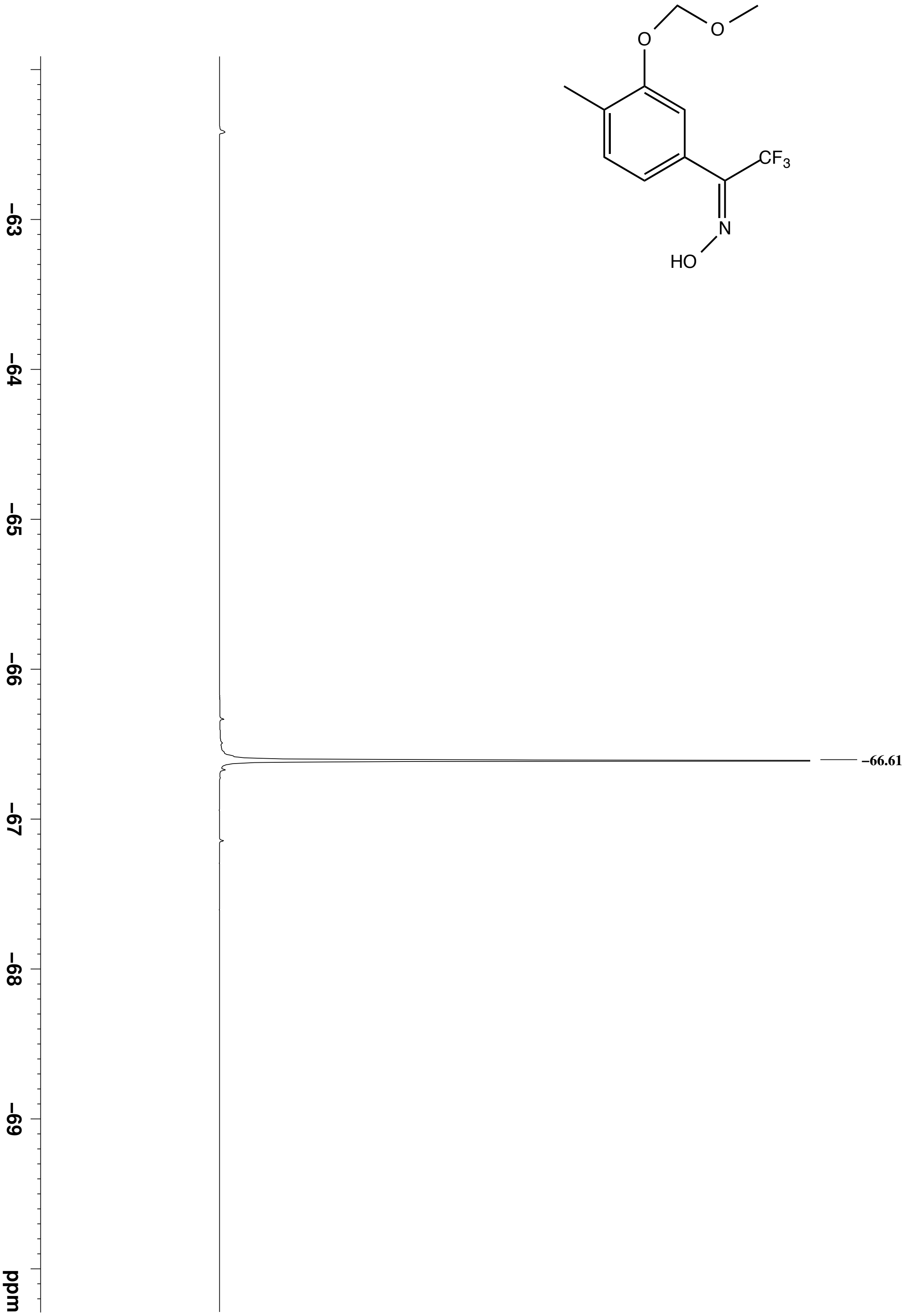

¹H NMR (500 MHz, $CDCl_3$) 2,2,2-trifluoro-1-(3-(methoxymethoxy)-4-methylphenyl)ethan-1-one O-tosyl oxime (5)

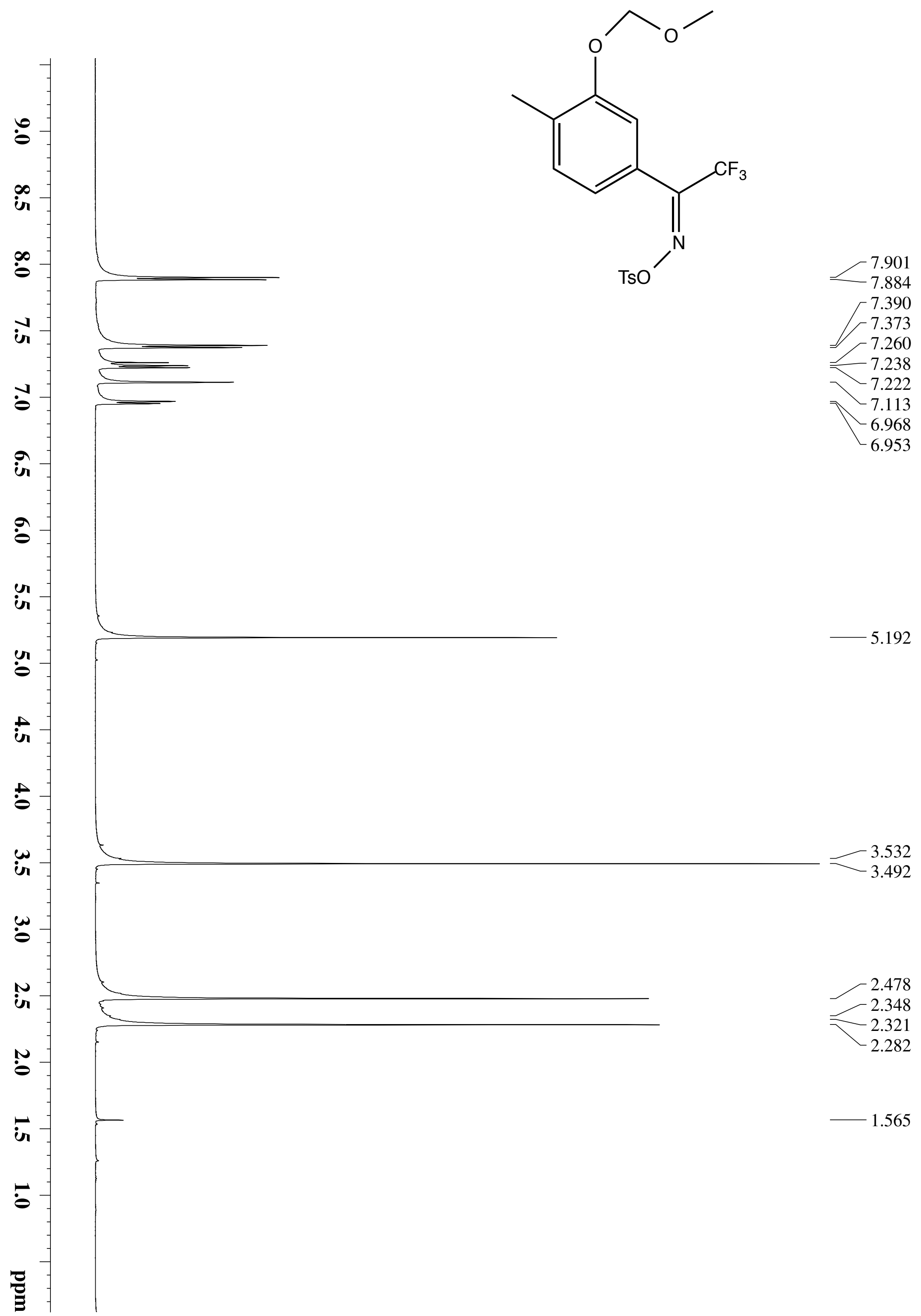

^{13}C NMR (500 MHz, $CDCl_3$) 2,2,2-trifluoro-1-(3-(methoxymethoxy)-4-methylphenyl)ethan-1-one O-tosyl oxime (5)

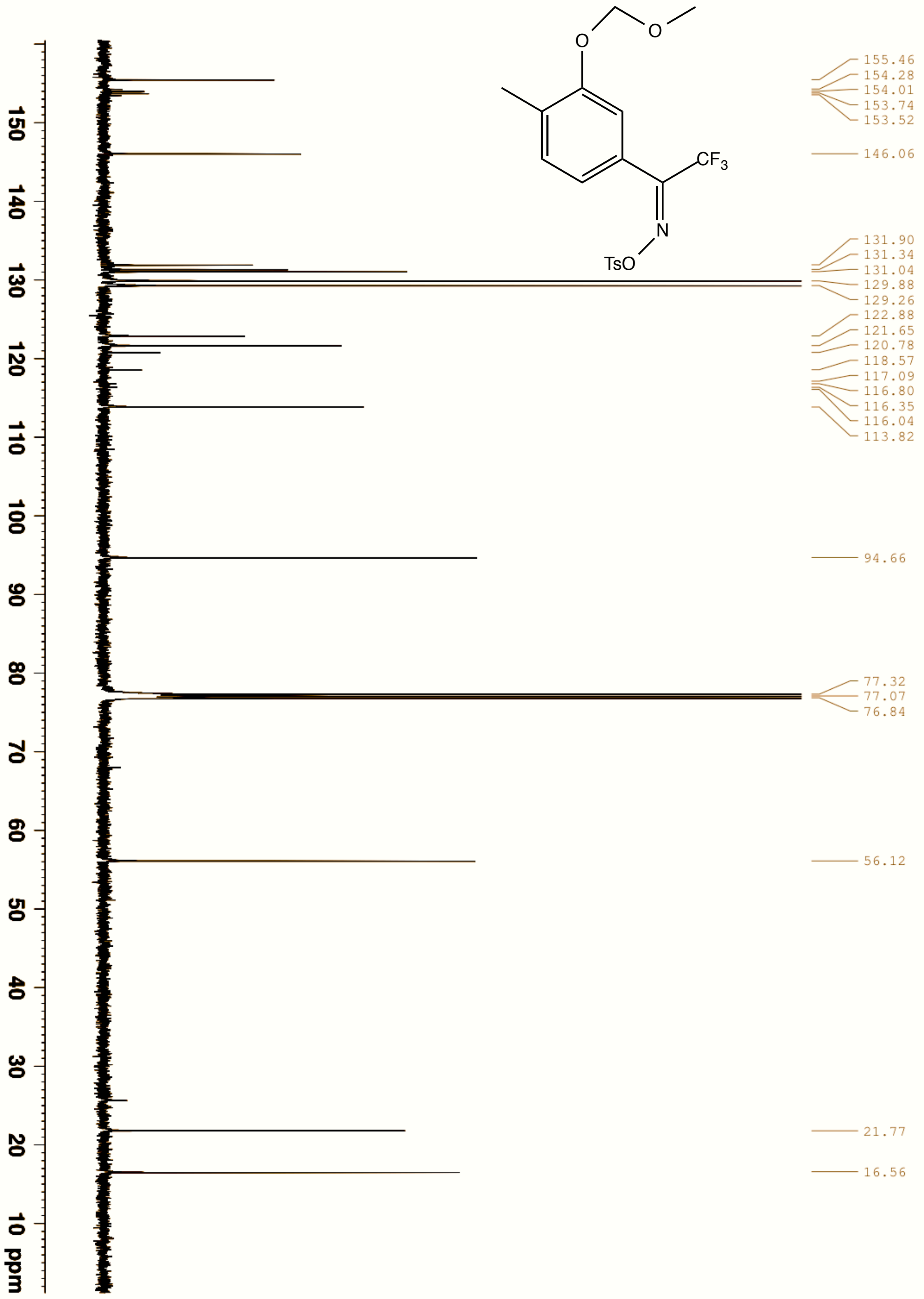

^{19}F NMR (360 MHz, $CDCl_3$) 2,2,2-trifluoro-1-(3-(methoxymethoxy)-4-methylphenyl)ethan-1-one O-tosyl oxime (5)

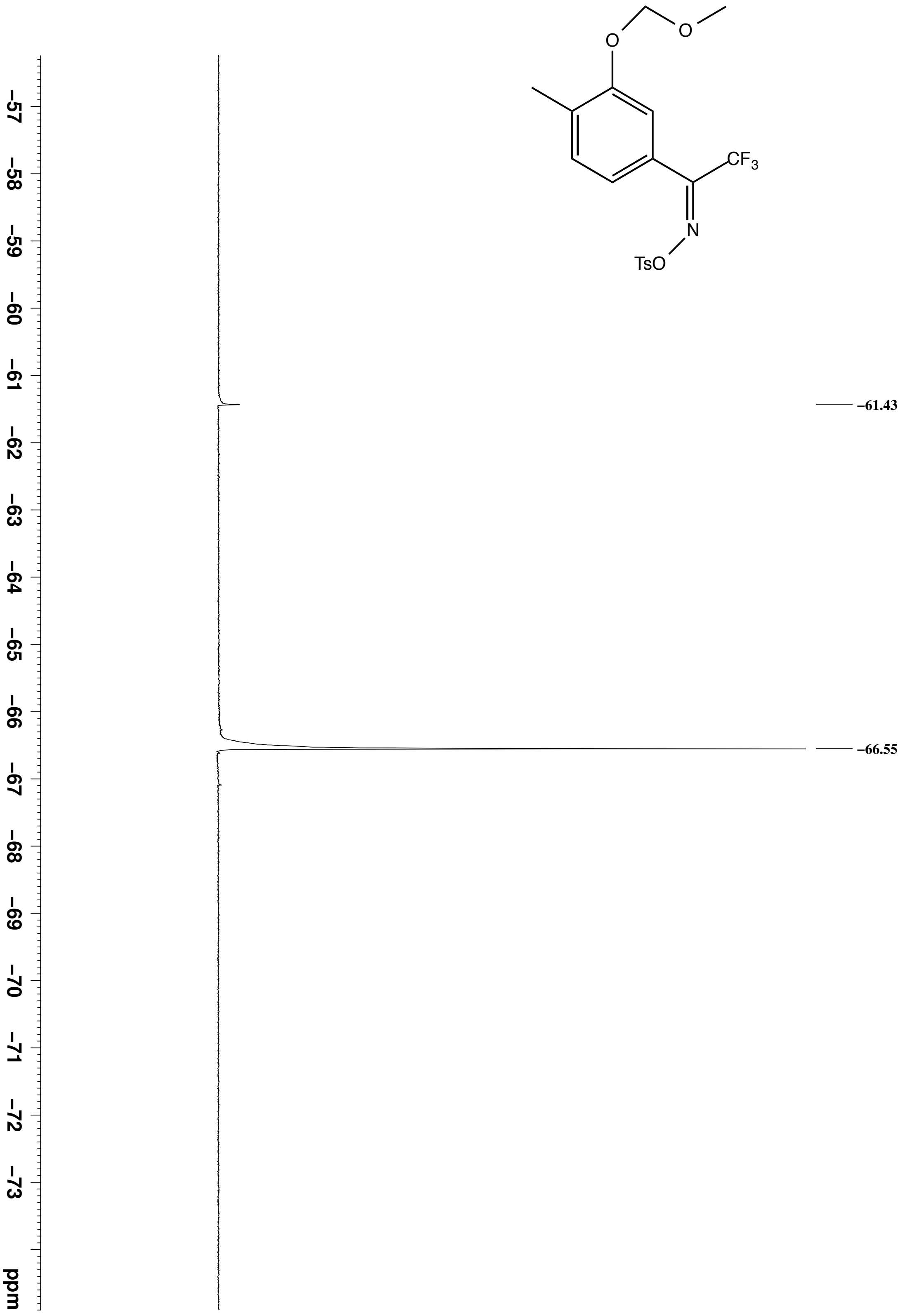

^{1}H NMR (500 MHz, $CDCl_3$) 3-(3-(methoxymethoxy)-4-methylphenyl)-3-(trifluoromethyl)diaziridine (6)

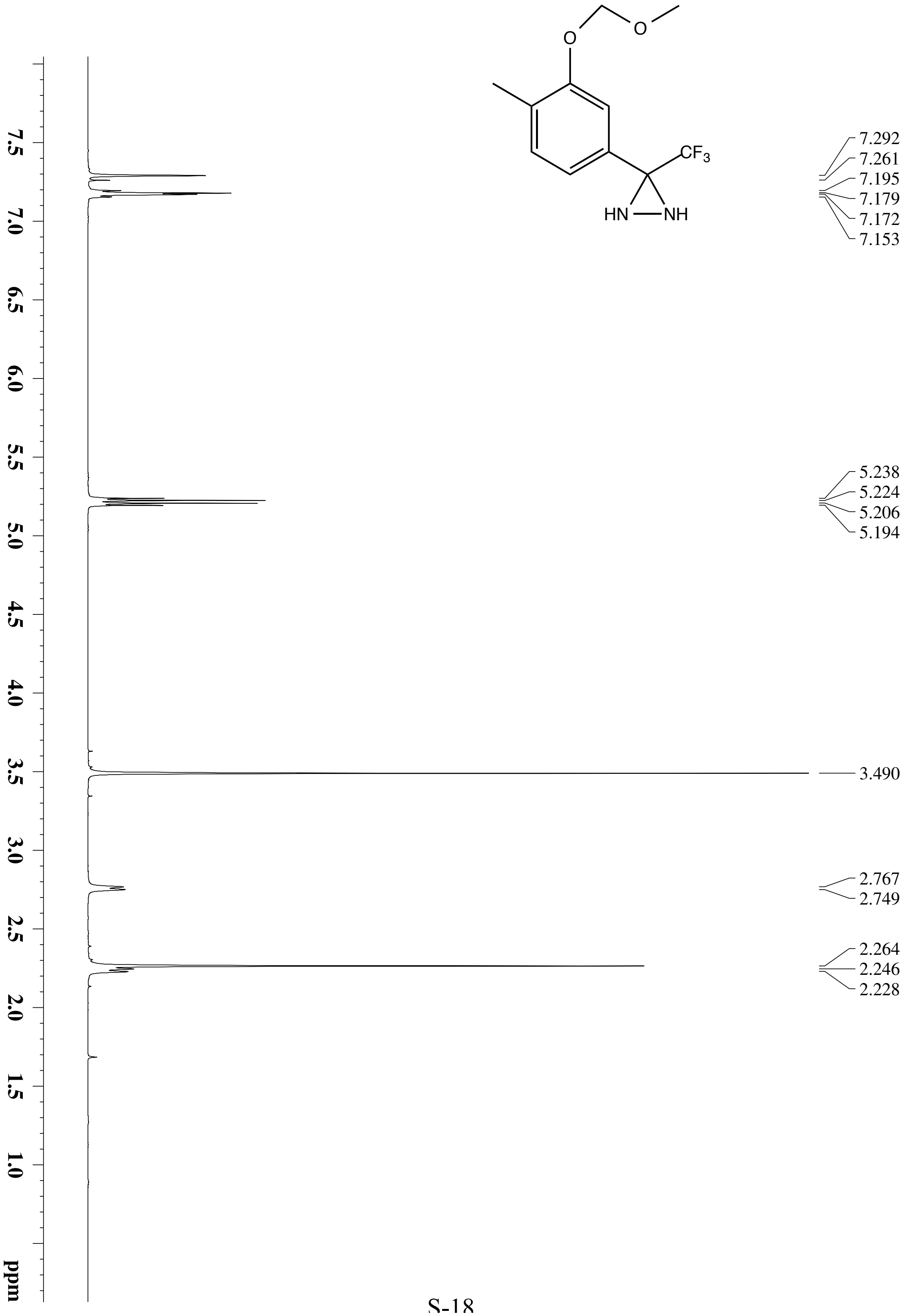

^{13}C NMR (500 MHz, $CDCl_3$) 3-(3-(methoxymethoxy)-4-methylphenyl)-3-(trifluoromethyl)diaziridine (6)

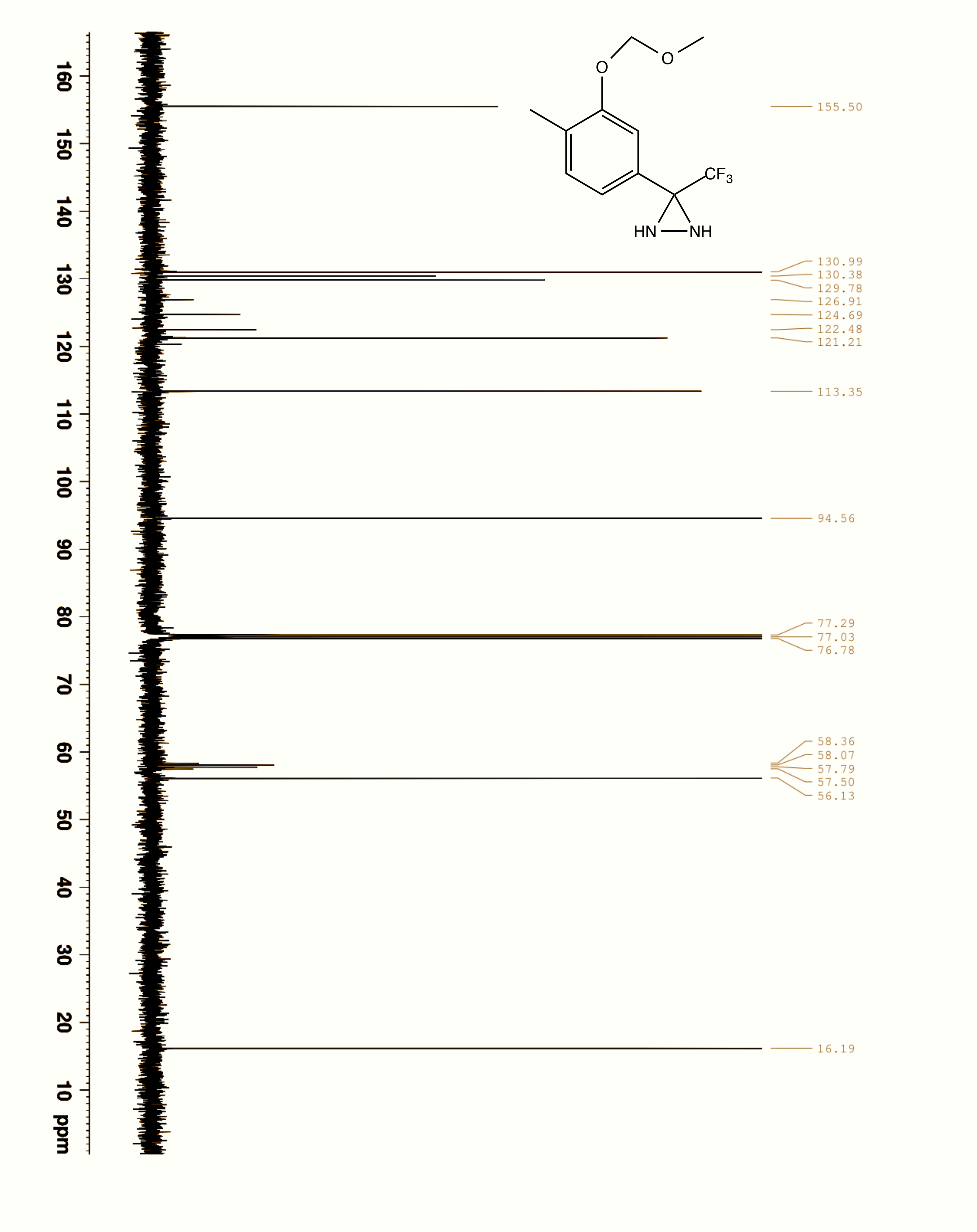

^{19}F NMR (360 MHz, $CDCl_3$) 3-(3-(methoxymethoxy)-4-methylphenyl)-3-(trifluoromethyl)diaziridine (6)

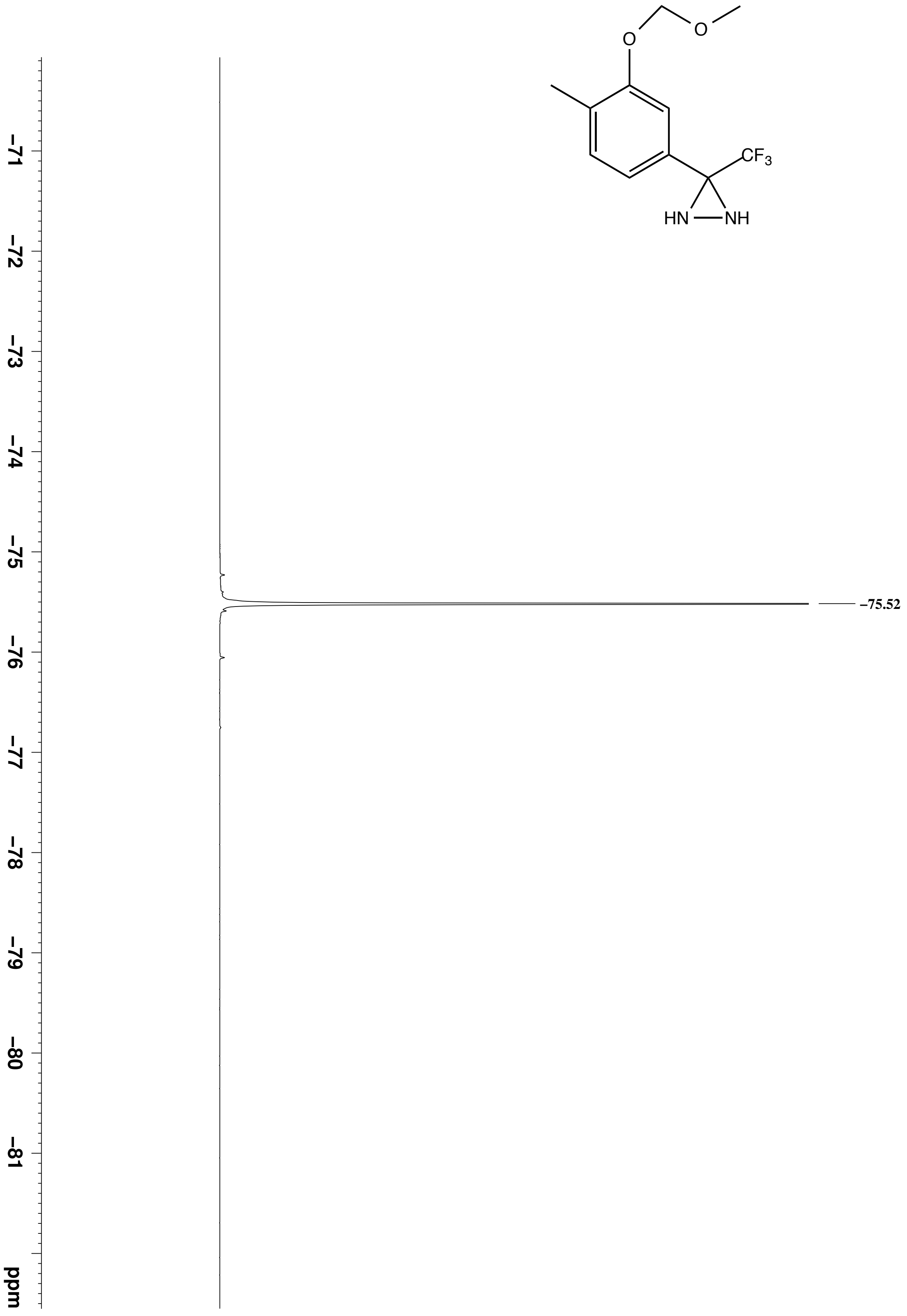

^{1}H NMR (500MHz, $CDCl_3$) 3-(3-(methoxymethoxy)-4-methylphenyl)-3-(trifluoromethyl) 3*H*-diazirine (7)

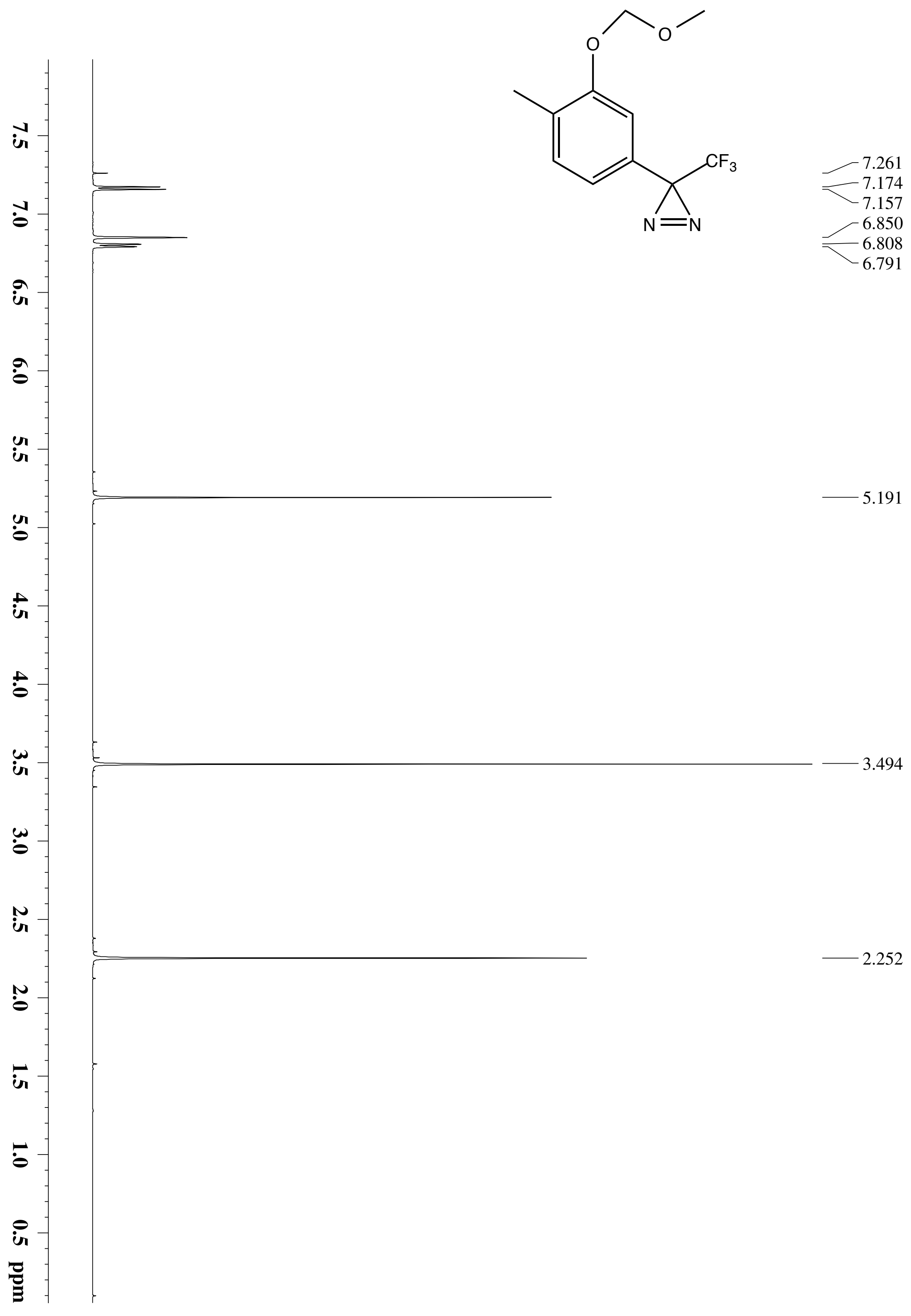

^{13}C NMR (500MHz, $CDCl_3$) 3-(3-(methoxymethoxy)-4-methylphenyl)-3-(trifluoromethyl) 3*H*-diazirine (7)

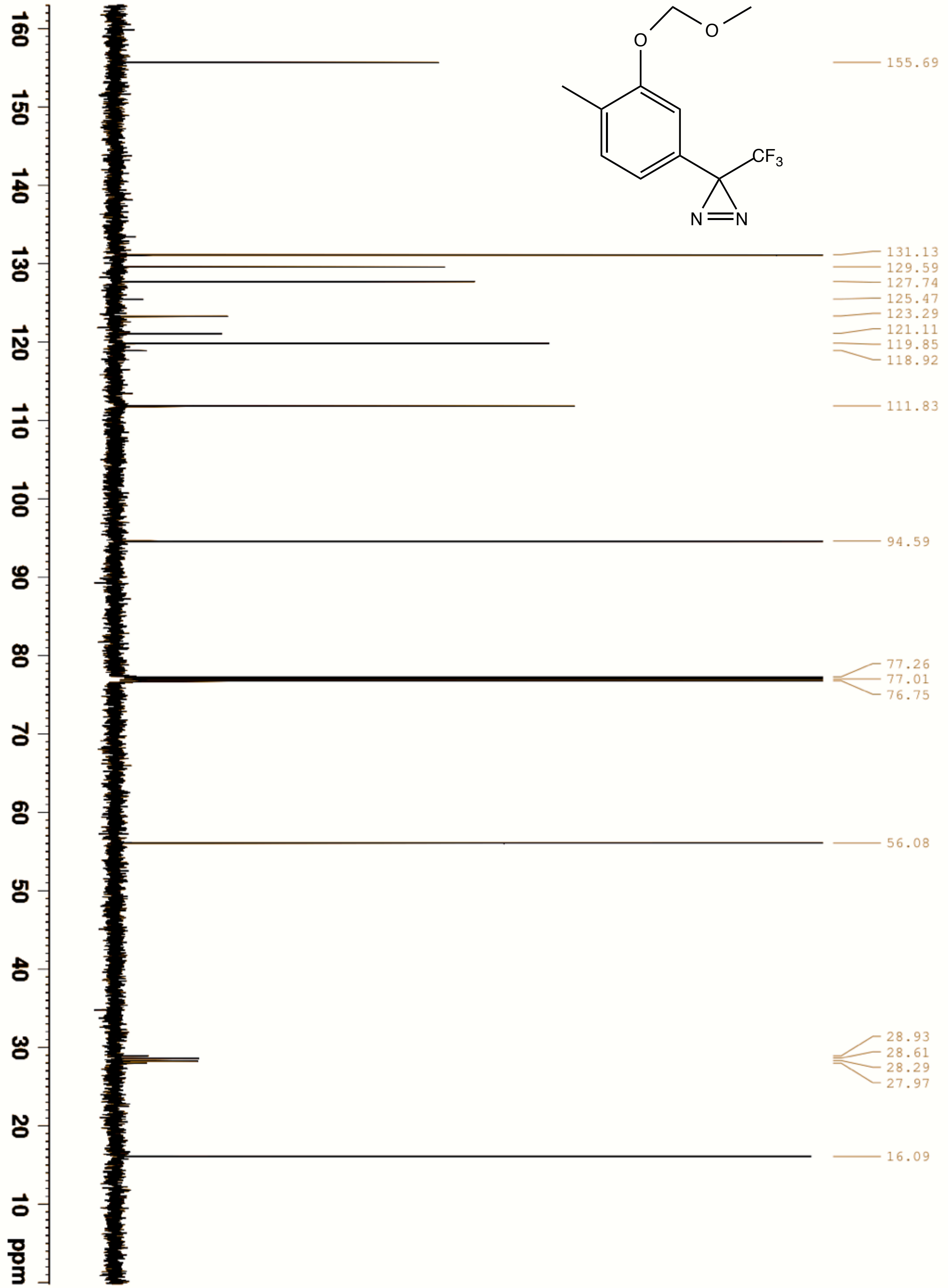

^{19}F NMR (360 MHz, $CDCl_3$) 3-(3-(methoxymethoxy)-4-methylphenyl)-3-(trifluoromethyl) 3*H*-diazirine (7)

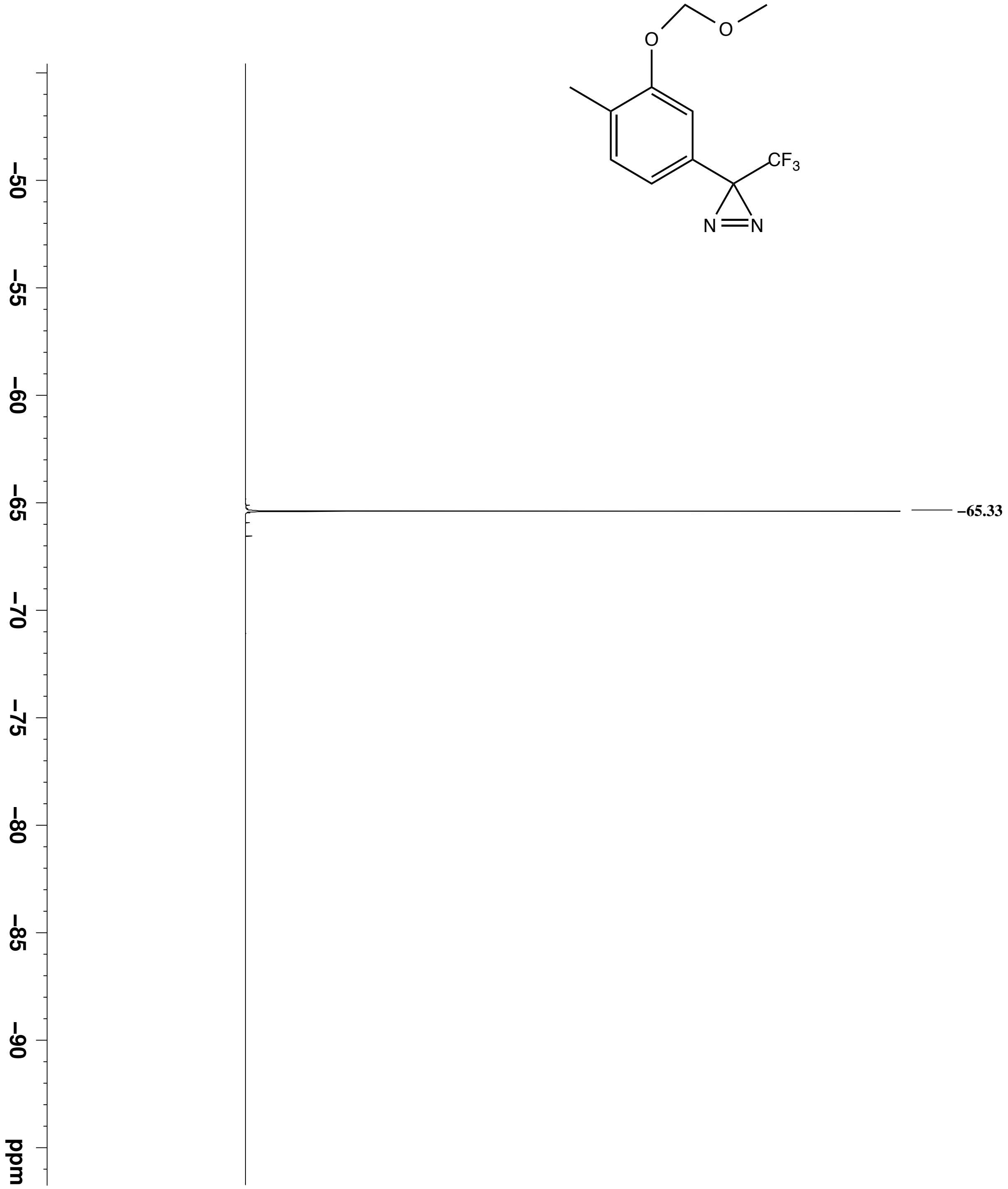

^{1}H NMR (500 MHz, $CDCl_3$) 3-(4-(bromomethyl)-3-(methoxymethoxy)phenyl)-3-(trifluoromethyl)-3*H*-diazirine (8)

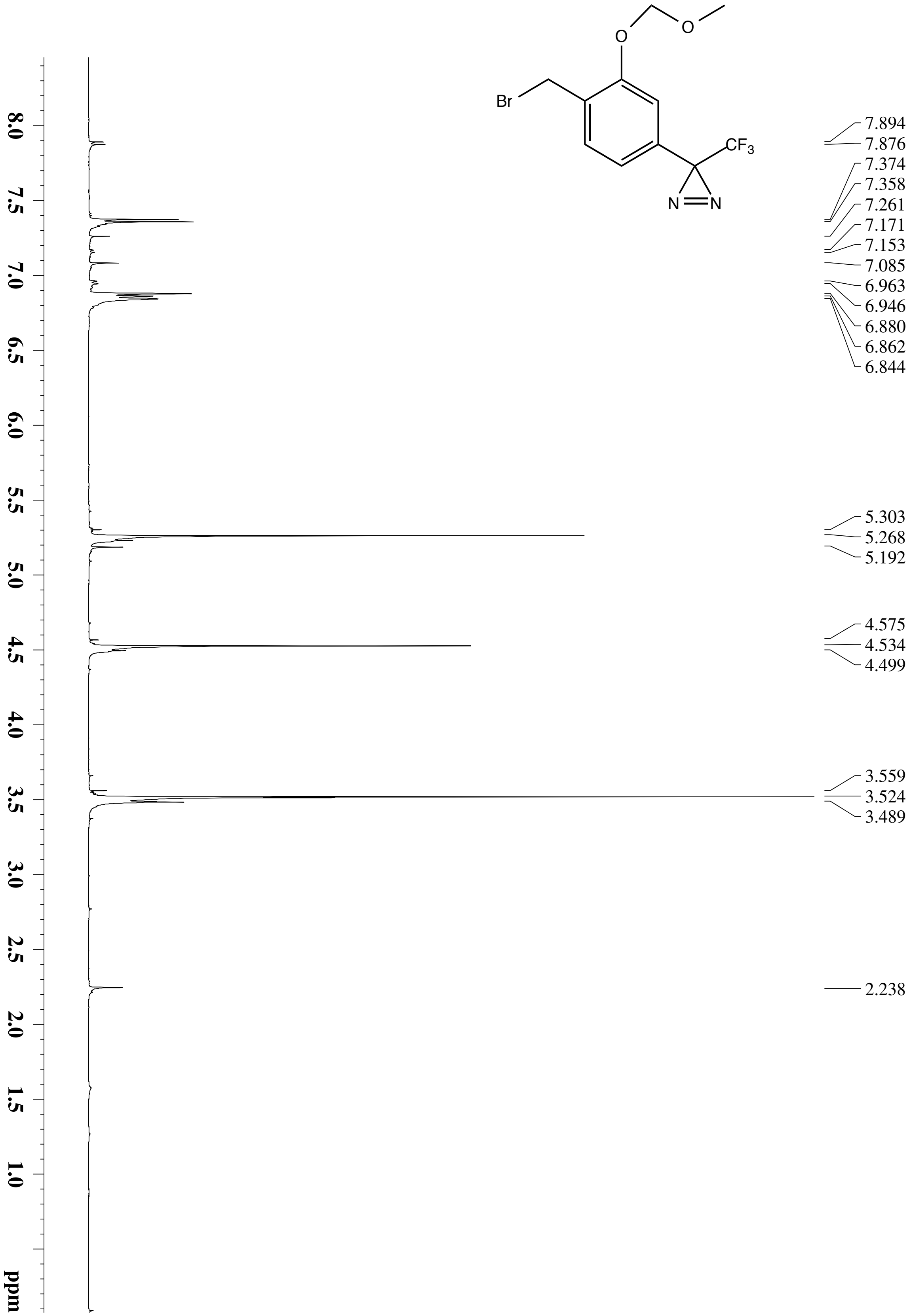

^{13}C NMR (500 MHz, $CDCl_3$) 3-(4-(bromomethyl)-3-(methoxymethoxy)phenyl)-3-(trifluoromethyl)-3*H*-diazirine (8)

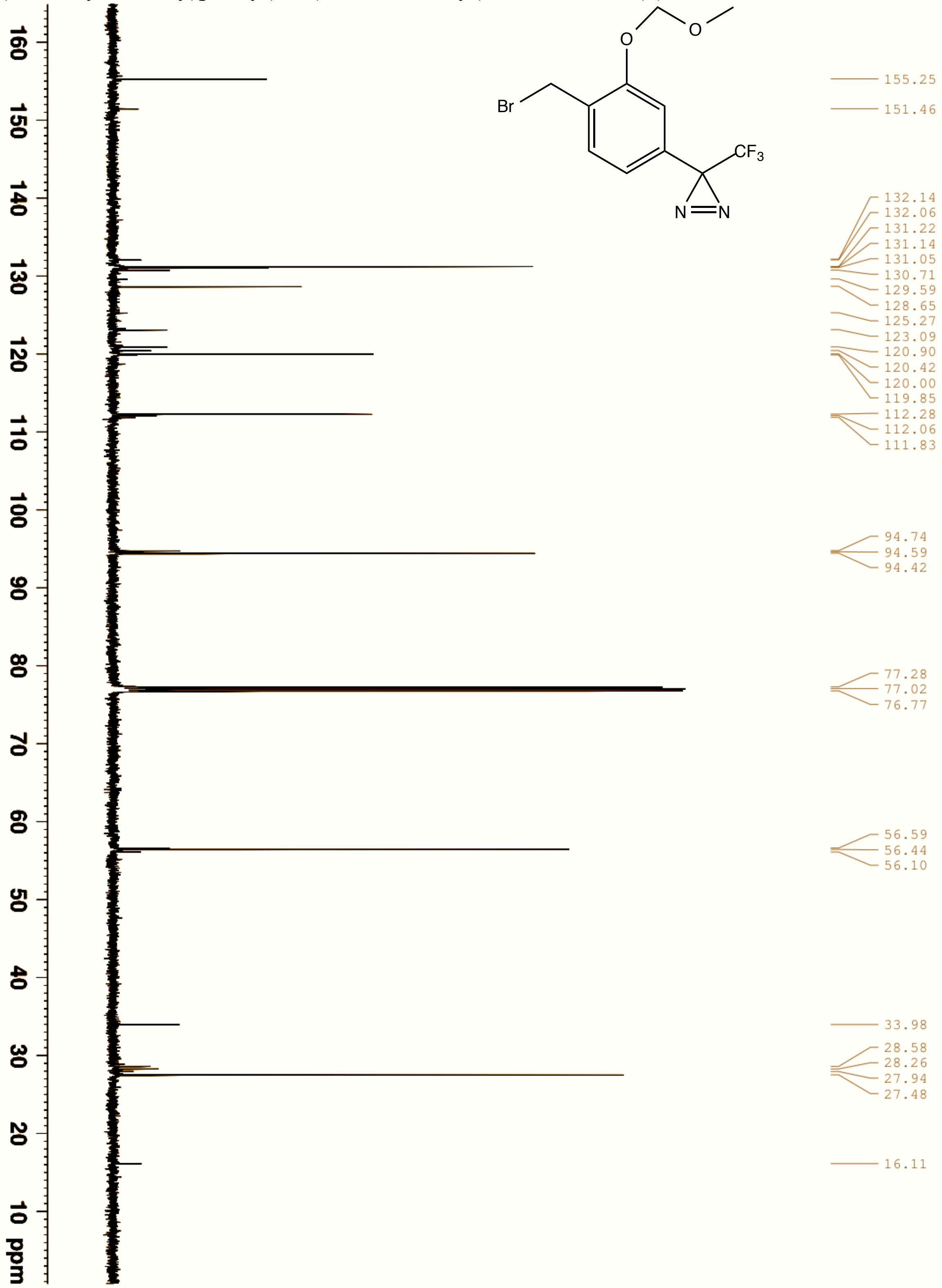

^{19}F NMR (360 MHz, $CDCl_3$) 3-(4-(bromomethyl)-3-(methoxymethoxy)phenyl)-3-(trifluoromethyl)-3*H*-diazirine (8)

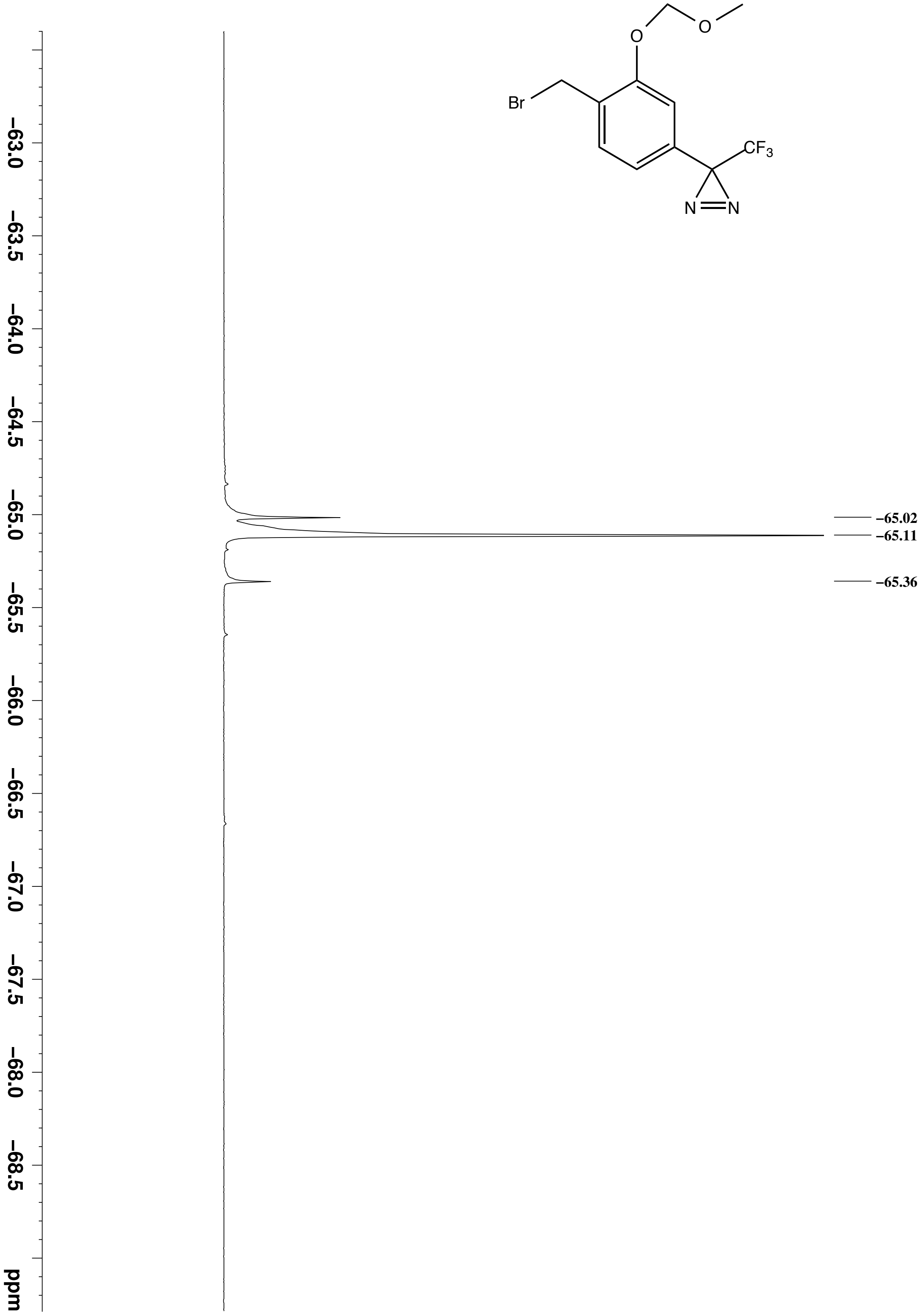

^{1}H NMR (500 MHz, $CDCl_3$) 3-(3-(methoxymethoxy)-4-((prop-2-yn-1-yloxy)methyl)phenyl)-3-(trifluoromethyl)-3*H*-diazirine (9)

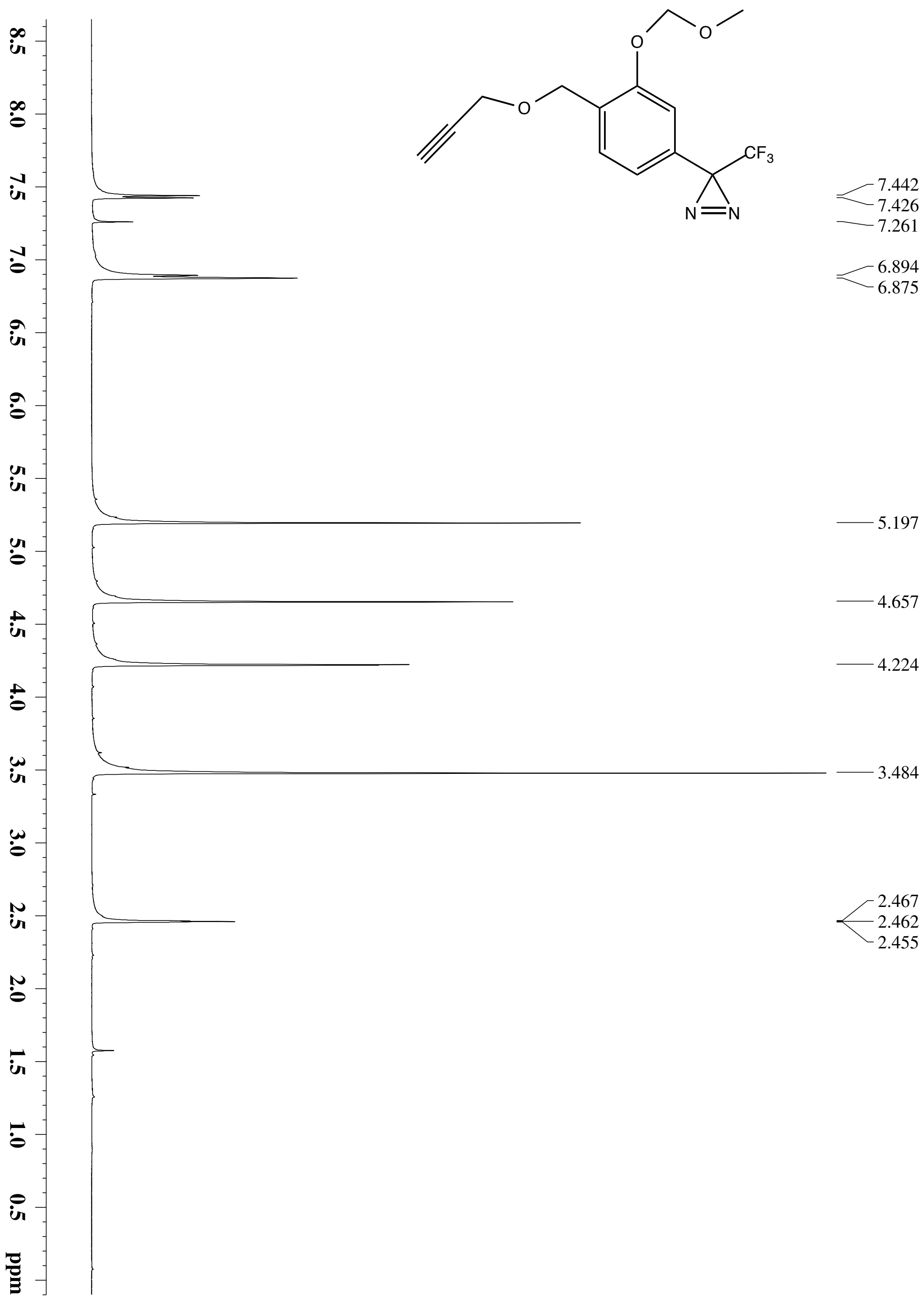

^{13}C NMR (500 MHz, $CDCl_3$) 3-(3-(methoxymethoxy)-4-((prop-2-yn-1-yloxy)methyl)phenyl)-3-(trifluoromethyl)-3*H*-diazirine (9)

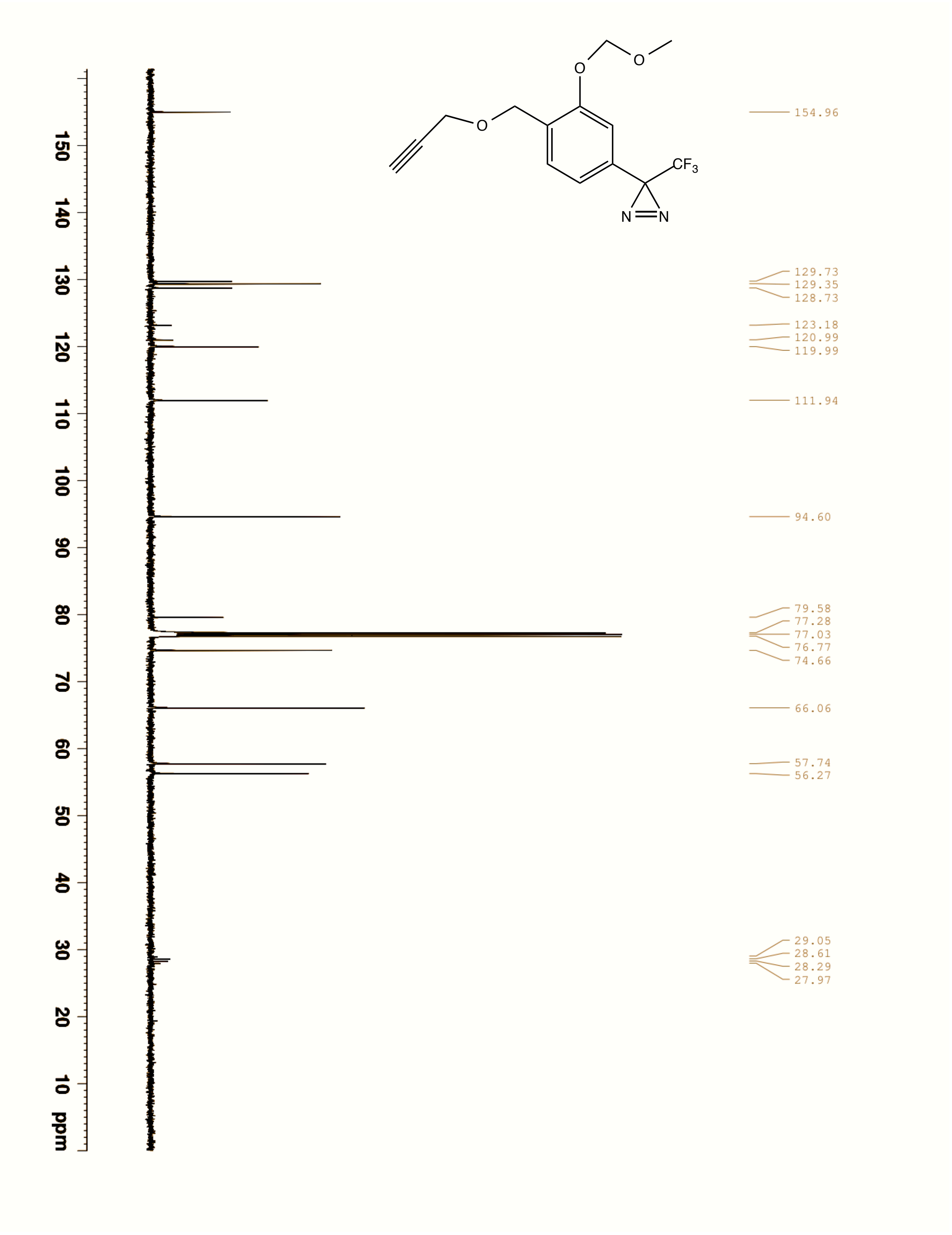

^{19}F NMR (360 MHz, $CDCl_3$) 3-(3-(methoxymethoxy)-4-((prop-2-yn-1-yloxy)methyl)phenyl)-3-(trifluoromethyl)-3*H*-diazirine (9)

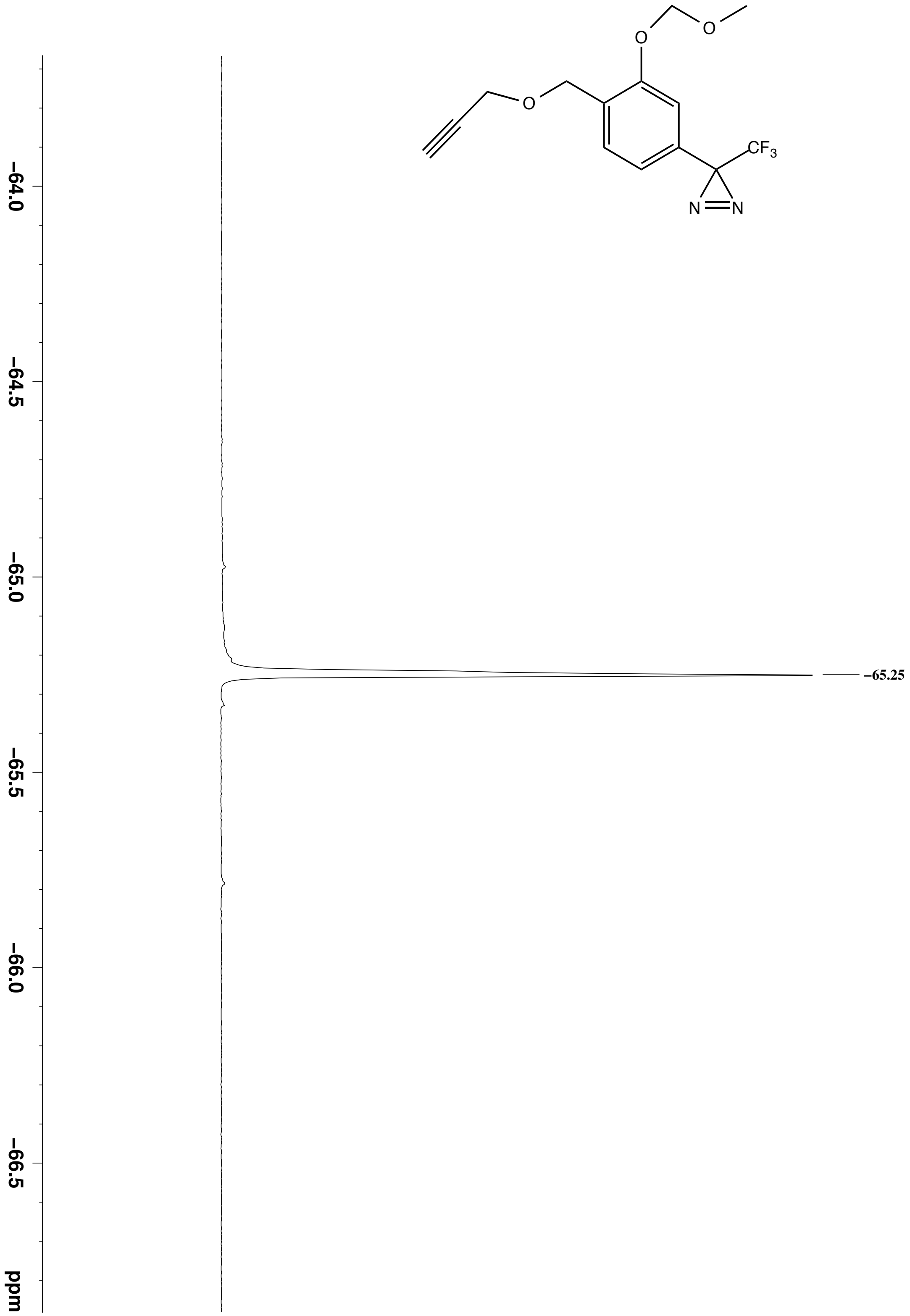

^{1}H NMR (500 MHz, $CDCl_3$) 2-((prop-2-yn-1-yloxy)methyl)-5-(3-(trifluoromethyl)-3*H*-diazirin-3-yl)phenol (1)

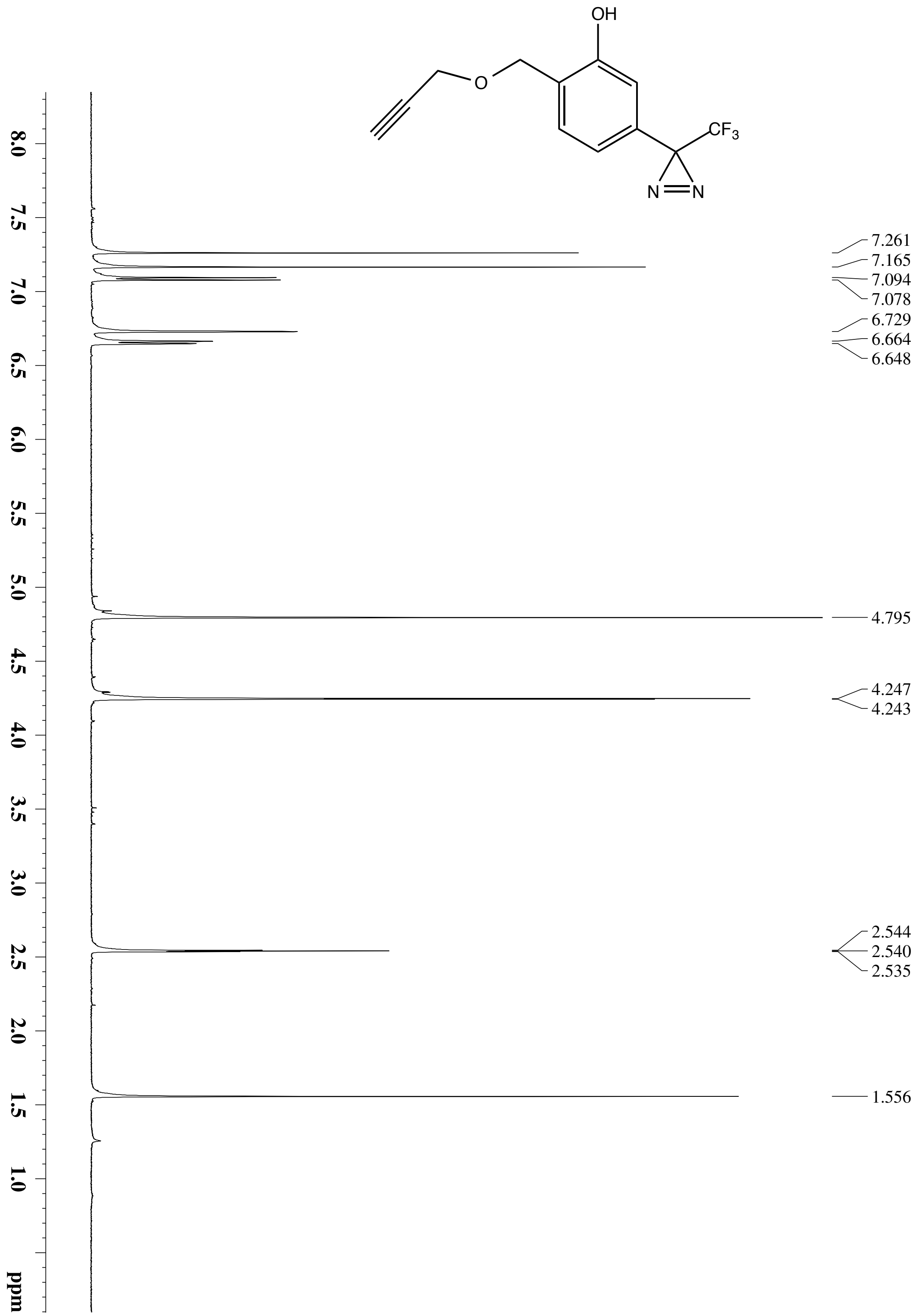

^{13}C NMR (500 MHz, $CDCl_3$) 2-((prop-2-yn-1-yloxy)methyl)-5-(3-(trifluoromethyl)-3*H*-diazirin-3-yl)phenol (1)

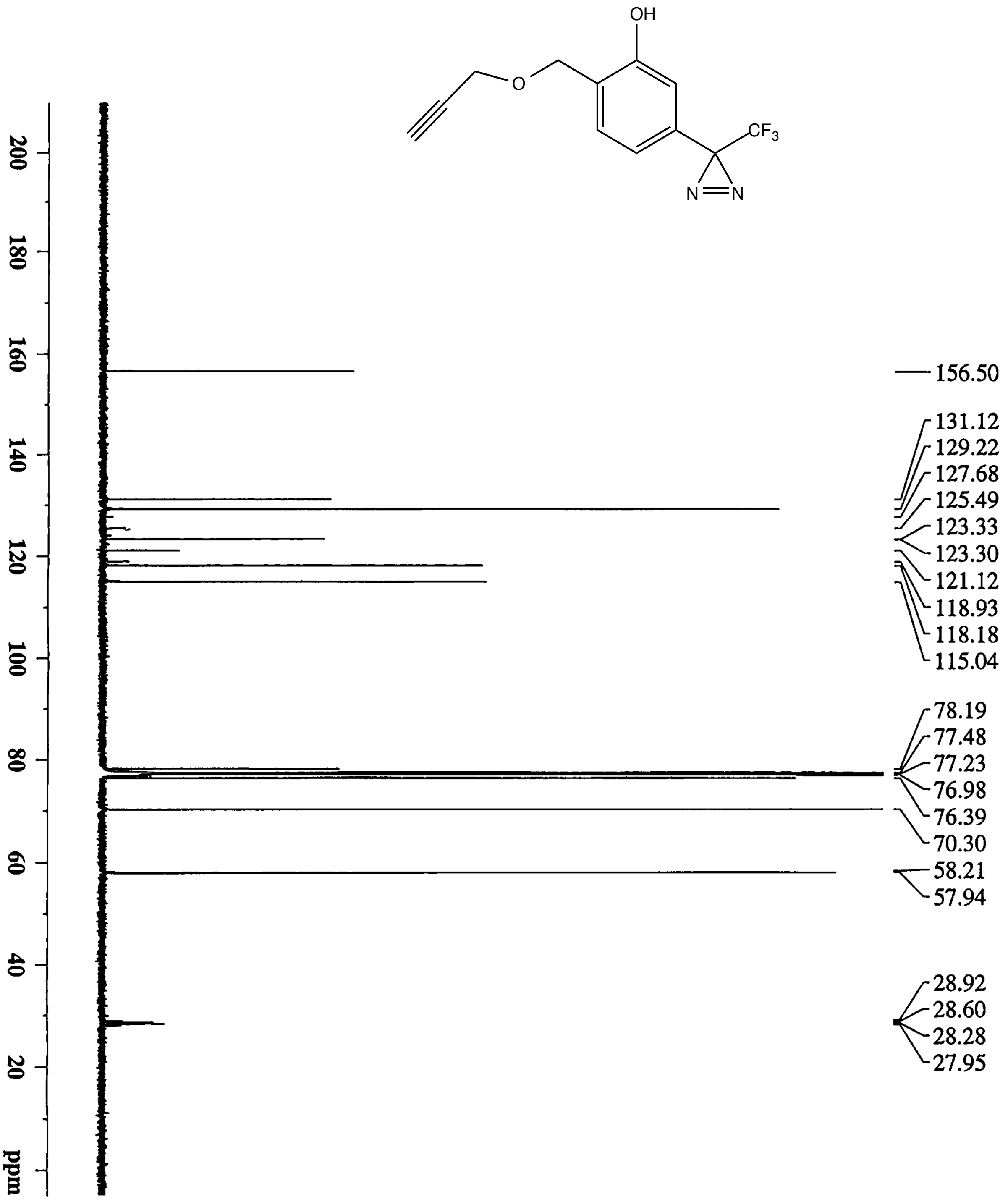

^{19}F NMR (360 MHz, $CDCl_3$) 2-((prop-2-yn-1-yloxy)methyl)-5-(3-(trifluoromethyl)-3*H*-diazirin-3-yl)phenol (1)

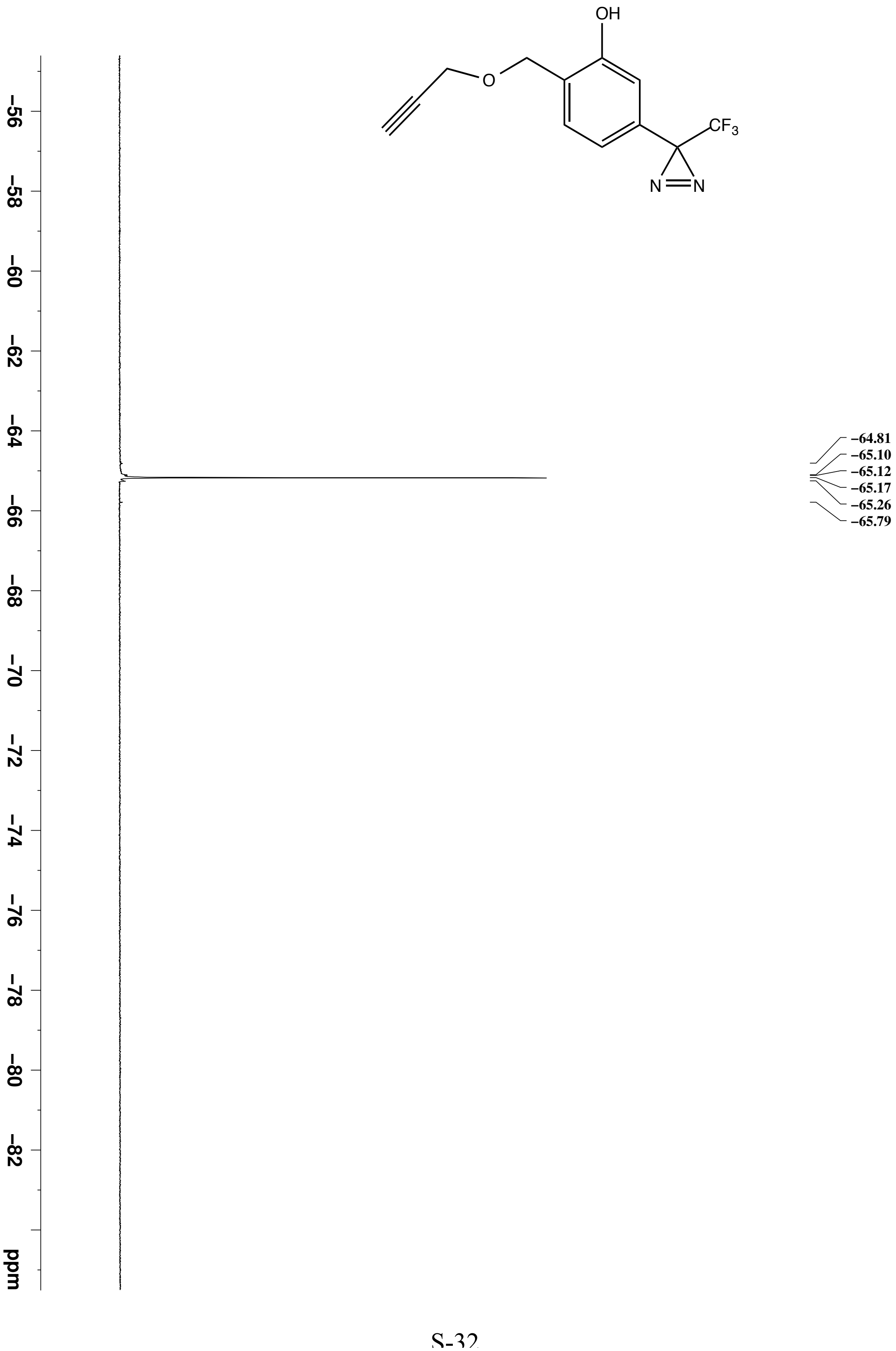

Supplement: Supplemental Data [file 10.1074_M116.736975_jbc.M116.736975-1.pdf]
